# Determination of Reaction Kinetics by Time‐Resolved Small‐Angle X‐ray Scattering during Polymerization‐Induced Self‐Assembly: Direct Evidence for Monomer‐Swollen Nanoparticles

**DOI:** 10.1002/anie.202312119

**Published:** 2023-12-06

**Authors:** Guoxing Liao, Matthew J. Derry, Andrew J. Smith, Steven P. Armes, Oleksandr O. Mykhaylyk

**Affiliations:** ^1^ Department of Chemistry University of Sheffield Dainton Building Sheffield S3 7HF UK; ^2^ South China Advanced Institute for Soft Matter Science and Technology School of Emergent Soft Matter Guangdong Provincial Key Laboratory of Functional and Intelligent Hybrid Materials and Devices South China University of Technology Guangzhou 510640 China; ^3^ Aston Advanced Materials Research Centre Aston University Aston Triangle Birmingham B4 7ET UK; ^4^ Beamline I22 Diamond Light Source Ltd Diamond House Didcot OX11 0DE UK

**Keywords:** Block Copolymer Nanoparticles, Polymerization Kinetics, Polymerization-Induced Self-Assembly, RAFT Dispersion Polymerization, Time-Resolved Small-Angle X-Ray Scattering

## Abstract

The kinetics of heterogeneous polymerization is determined directly using small‐angle X‐ray scattering (SAXS). This important advancement is exemplified for the synthesis of sterically‐stabilized diblock copolymer nanoparticles by reversible addition‐fragmentation chain transfer (RAFT) dispersion polymerization of benzyl methacrylate (BzMA) in mineral oil at 90 °C. The principle of mass balance is invoked to derive a series of equations for the analysis of the resulting time‐resolved SAXS patterns. Importantly, there is a continuous change in the X‐ray scattering length density for the various components within the reaction mixture. This enables the volume fraction of unreacted BzMA monomer to be calculated at any given time point, which enables the polymerization kinetics to be monitored in situ directly without relying on supplementary characterization techniques. Moreover, SAXS enables the *local* concentration of both monomer and solvent within the growing swollen nanoparticles to be determined during the polymerization. Data analysis reveals that the instantaneous rate of BzMA polymerization is proportional to the local monomer concentration within the nanoparticles. In principle, this powerful new time‐resolved SAXS approach can be applicable to other heterogeneous polymerization formulations.

## Graphical abstract

A generic SAXS model for polymerization‐induced self‐assembly (PISA) formulations has been developed by applying the law of conservation of mass. Time‐resolved small‐angle X‐ray scattering (SAXS) data are analyzed to determine the kinetics of polymerization directly during the synthesis of spherical nanoparticles. This new analytical approach enables calculation of the mass transport of monomer and solvent in and out of the growing nanoparticles.

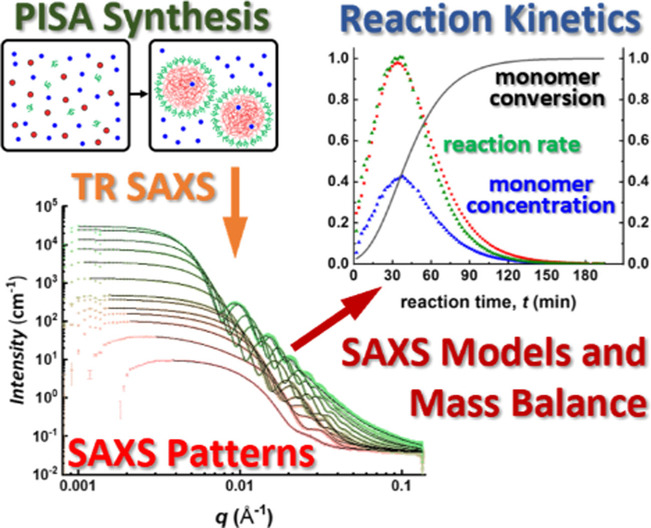

## Introduction

It is widely recognized that block copolymer self‐assembly offers potential applications in cell biology,^[1]^ templating,^[2]^ sensors,^[3]^ nanolithography[^3a^, ^4^] and other technologies.^[5]^ One of the most convenient and versatile chemistries for the synthesis of well‐defined functional block copolymers is reversible addition‐fragmentation chain transfer (RAFT) polymerization.^[6]^ This approach provides good control over the target degree of polymerization (DP) and the molecular weight distribution.^[7]^ Traditional post‐polymerization processing routes to block copolymer nano‐objects are typically only conducted at rather low copolymer concentrations (<1% w/w).^[8]^ Fortunately, the development of RAFT‐mediated polymerization‐induced self‐assembly (PISA) provides convenient access to a wide range of block copolymer nano‐objects at relatively high solids (25–50 % w/w).^[9]^ Moreover, the latter method is suitable for a wide range of media, including polar solvents, non‐polar solvents and ionic liquids.[^9b^, ^10^] In particular, PISA syntheses via RAFT dispersion polymerization involves chain extension of a solvophilic stabilizer block using a miscible monomer to produce a solvophobic structure‐directing block.[^9b^, ^10a^] Once a critical chain length has been achieved, micellar nucleation occurs and the growing diblock copolymer chains self‐assemble to form spherical nuclei.[^9b^, ^9c^, ^10a^, ^11^] Under certain well‐established conditions, the copolymer morphology can subsequently evolve to produce sterically‐stabilized worms, vesicles, multilamellar vesicles or lamellae.[^9b^, ^10a^] Alternatively, the nascent nuclei can simply grow in size to produce kinetically‐trapped spheres.[^9f^, ^12^] Clearly, RAFT dispersion polymerization is a complex multistage process. It is well‐known that the in situ self‐assembly of the growing diblock copolymer chains that occurs during such heterogeneous PISA formulations strongly influences the reaction kinetics.[^9b^, ^9k^, ^10a^, ^13^]

^1^H NMR spectroscopy is widely used to determine monomer conversion during PISA syntheses[^9b^, ^10a^] and in situ experiments have been performed in a few cases.[^7b^, ^14^] Although this technique is useful for monitoring the monomer concentration, it cannot provide any information regarding the evolution in particle size and morphology. However, such studies are crucial because they enable rigorous characterization of the self‐assembled nanoparticles that act as individual nanoreactors during PISA. In principle, dynamic light scattering (DLS) can offer useful insights, at least for kinetically‐trapped spheres. Indeed, periodic sampling of polymerizing reaction mixtures followed by ex situ DLS analysis has been conducted in various studies.[^9j^, ^11^, ^13a^, ^15^, ^16^] However, conventional DLS instruments cannot be used to monitor concentrated colloidal dispersions in situ.^[17]^ Moreover, DLS provides only very limited structural information for higher order morphologies such as worms and vesicles. As an alternative to DLS, transmission electron microscopy (TEM) is widely used to assign copolymer morphologies but this technique usually suffers from relatively poor statistics and can be prone to drying artefacts.[^8a^, ^9b^, ^10a^] To overcome the latter problem, cryo‐TEM studies have been undertaken for selected PISA formulations. However, in at least some cases there is insufficient contrast between the nano‐objects and the frozen aqueous matrix.^[18]^ Recently, a liquid cell has been developed for in situ TEM studies during PISA.^[19]^ Unfortunately, imaging can only be performed under certain conditions because radicals generated by the electron beam can initiate the polymerization. In principle, using a lower energy electron beam can reduce this problem and minimize beam damage but in practice this approach invariably leads to lower resolution.^[20]^

**Scheme 1:**
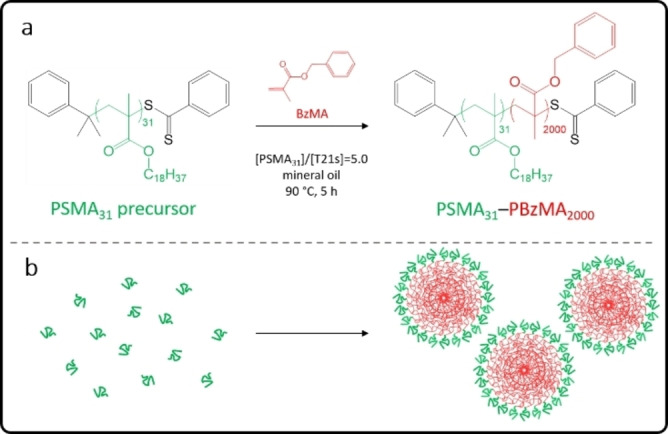
(a) Synthesis of poly(stearyl methacrylate)‐poly(benzyl methacrylate) (PSMA_31_–PBzMA_2000_) spherical nanoparticles via chain extension of a PSMA_31_ precursor by RAFT dispersion polymerization of BzMA in mineral oil at 90 °C. (b) Schematic representation of the self‐assembly that occurs when growing an oil‐insoluble PBzMA chain from an oil‐soluble PSMA_31_ precursor to produce sterically‐stabilized nanoparticles (or micelles).

Over the past decade, small‐angle X‐ray scattering (SAXS) has been used to characterize block copolymer spheres, worms, vesicles and framboidal vesicles prepared via PISA in various media.[^11^, ^13c^, ^14c^, ^15e^, ^21^] Moreover, this powerful structural technique also enables the in situ evolution in copolymer morphology to be monitored during PISA.[^13b^, ^14c^, ^22^] For example, Derry and co‐workers employed time‐resolved SAXS (TR‐SAXS) combined with ex situ ^1^H NMR spectroscopy studies to examine the RAFT dispersion polymerization of benzyl methacrylate (BzMA) at 90 °C in mineral oil using a poly(stearyl methacrylate) (PSMA) precursor (see Scheme 1).^[13c]^ Depending on the mean DP of the PSMA stabilizer block, the final copolymer morphology was either vesicles or kinetically‐trapped spheres.^[13c]^ Unfortunately, the high‐energy synchrotron X‐ray beam generated a radical flux in addition to that provided by the initiator, which meant that the rate of polymerization within the capillary sample holder was significantly faster than that observed for the equivalent synthesis conducted in the absence of any X‐ray flux. Thus, ^1^H NMR kinetic data obtained for a laboratory‐scale synthesis conducted under the latter conditions could not be used to determine the instantaneous monomer conversion for each SAXS pattern. To address this problem, the NMR kinetic data were renormalized to the much shorter timescale indicated by the SAXS experiments. Subsequently, close reference to the phase boundaries observed within the relevant pseudo‐phase diagram constructed for a series of such diblock copolymer nano‐objects indicated that such renormalization was a reasonable approach.^[13c]^ Accordingly, SAXS patterns were analyzed using appropriate scattering models but satisfactory data fits required further information regarding the degree of solvation of the insoluble poly(benzyl methacrylate) (PBzMA) chains by BzMA monomer, as well as the extent of solvent plasticization by the mineral oil. In the present study, this particular PISA formulation is revisited and the original SAXS patterns are subjected to a much more powerful and rigorous SAXS analysis. This new analytical approach reveals fundamental new insights regarding the true nature of PISA.

For dispersion polymerization formulations, the monomer should be fully miscible with the solvent. Hence the initial reaction solution comprises a binary mixture of monomer and mineral oil that has a mean X‐ray scattering length density (SLD) which lies between the SLD of each pure component. As the polymerization proceeds, monomer is converted into polymer and the binary monomer/solvent composition gradually shifts towards that of the pure solvent. If this system remained homogeneous, SAXS studies would not be particularly informative. However, micellar nucleation results in the formation of nascent nanoparticles (see Scheme 1) and the growing insoluble chains within such nuclei quickly become solvated by unreacted monomer. Indeed, such monomer solvation is essential for the polymerization to continue and often leads to a significant rate enhancement owing to the relatively high local monomer concentration within the nanoparticles compared to that within the continuous phase.[^9c^, ^13c^] Importantly, such solvation necessarily also lowers the effective X‐ray SLD for the nascent nanoparticle cores. Prior to the present study, this subtle point appears to be under‐appreciated in the literature. More specifically, if a distinct interface develops between the locus of polymerization and the continuous phase, this should enable calculation of both the *instantaneous* monomer concentration and the *local* monomer concentration within the growing nanoparticles, which in turn provides useful information regarding the mass transport of monomer and the ensuing rate of polymerization.

Herein a generic SAXS model for RAFT dispersion polymerization formulations has been developed by applying the law of conservation of mass to the oil‐soluble homopolymer precursor, the BzMA monomer and the final diblock copolymer chains. In particular, the TR‐SAXS data reported by Derry and co‐workers^[13c]^ have been reanalyzed to determine the kinetics of polymerization directly during the synthesis of PSMA_31_‐PBzMA_2000_ spherical nanoparticles. This new analytical approach enables calculation of the instantaneous mean DP of the growing PBzMA chains during the evolution in copolymer morphology. Moreover, the results provide important new information regarding the change in local monomer concentration within the growing nanoparticles over time and their degree of solvent plasticization, as well as the evolution in particle size and aggregation number. Accordingly, valuable new insights can be obtained regarding the RAFT polymerization kinetics, the rate of formation of block copolymer nanoparticles and their growth mechanism. In principle, this powerful strategy can be applicable to any dispersion polymerization formulation regardless of the solvent composition.

## Results and Discussion

**Figure 1:**
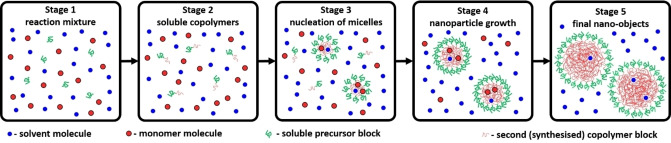
Schematic representation of a PISA synthesis for which the final copolymer morphology is kinetically‐trapped spheres. **Stage 1**: Initial reaction mixture comprising solvent molecules (small blue spheres), a soluble precursor or ‘macro‐CTA’ (green curves) and monomer molecules (small red spheres) that will polymerize to form the second block. **Stage 2**: Soluble block copolymer chains comprising a relatively short second block (red curves).^[9b]^ **Stage 3**: Nucleation of micelles with the growing insoluble second block with such chains located within the micelle cores (large red spheres). **Stage 4**: Nanoparticle growth. **Stage 5**: Final spherical diblock copolymer nanoparticles.

According to prior TEM and ^1^H NMR spectroscopy studies,[^9b^, ^9c^] the PISA synthesis of diblock copolymer nano‐objects via RAFT dispersion polymerization comprises at least five stages (Figure 1). **Stage 1** involves the preparation of a reaction mixture consisting of the solvent, the soluble precursor or steric stabilizer block (sometimes denoted as the ‘macro‐CTA’), and a suitable missible monomer for producing the insoluble structure‐directing block. Each of these components is uniformely distributed within the reaction solution. During **Stage 2**, some of the monomer is converted into the second block but at this early stage these growing chains are too short to cause nucleation and hence remain soluble within the monomer/solvent binary mixture. Once the growing second block reaches its critical DP (**Stage 3**), nucleation occurs and the diblock copolymer chains undergo spontaneous self‐assembly to form nascent micelles. Thus, the reaction mixture becomes heterogeneous. At this point, unreacted monomer is preferentially located within the micelles because it can solvate (or plasticize) the growing insoluble block. In contrast, the solvent is a poor solvent for such chains and hence is expected to be (at least partially) excluded from the micelle cores. Since the local concentration of the unreacted monomer within the micelles is higher than that in the continuous phase, a pronounced rate acceleration is usually observed immediately following micellar nucleation (**Stage 4**).^[9c]^ At this stage, the reaction takes place mainly in the self‐assembled micelles, which act as individual nanoreactors. As the monomer within the micelle cores is consumed, it is replenished via diffusion of further monomer from the continuous phase.^[23]^ Thus, a relatively fast rate of polymerization within the micelles is maintained and is most likely diffusion‐controlled. Once all the unreacted monomer is consumed, polymerization ceases and the final diblock copolymer nano‐objects are obtained (**Stage 5**). If the soluble precursor block is relatively long, then only kinetically‐trapped spheres are obtained via PISA.^[13c]^ This is because steric stabilization is more effective and hence prevents sphere‐sphere fusion, which is the critical first step for the formation of higher order morphologies (e.g. worms or vesicles). On the other hand, if the soluble precursor block is relatively short and the growing insoluble chains are sufficiently mobile, then the copolymer morphology can evolve into either worms (via stochastic 1D fusion of multiple spheres)^[15c]^ or vesicles (via a series of intermediate species that include worms, branched worms, octopuses and jellyfish) depending on the final DP targeted for the insoluble block.[^9c^, ^10a^, ^21d^] Given the complexity of the analysis, PISA syntheses that produce only kinetically‐trapped spheres were chosen for this initial proof‐of‐concept study. More specifically, the RAFT dispersion polymerization of BzMA in mineral oil at 90 °C using a PSMA_31_ precursor has been examined. For such PISA formulations, a sophisticated structural model has been constructed for the analysis of TR‐SAXS data recorded during the BzMA polymerization (equations S1–S31). ^1^H NMR spectroscopy analysis confirmed that virtually all of the BzMA monomer is converted into PBzMA during PISA. Moreover, TEM and DLS studies^[13c]^ indicated that the final diblock copolymer nano‐objects had a well‐defined kinetically‐trapped spherical morphology (see **Stage 5** in Figure 1). Furthermore, there was no significant difference between the last few SAXS patterns, indicating cessation of the BzMA polymerization within the experimental timeframe of 194 min. Thus, to a good approximation, the final frame of the time‐resolved SAXS data recorded after 194 min corresponds to PSMA_31_‐PBzMA_2000_ spherical nanoparticles. This assumption enabled the new SAXS model to be verified first on the known (final) product and then effectively applied frame‐by‐frame backwards from the final frame to the beginning of the reaction. Eq S2 can be combined with equations S31 and S32. Thus, the total scattering intensity for the final frame, as well as all other frames associated with **Stage 4** of this PISA synthesis (Figure 1), can be expressed as

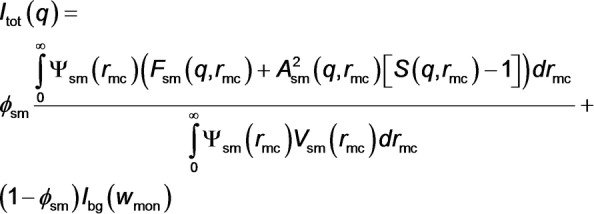




A full description of all the parameters given in eq 1 can be found in the **Supporting Information**.

**Figure 2:**
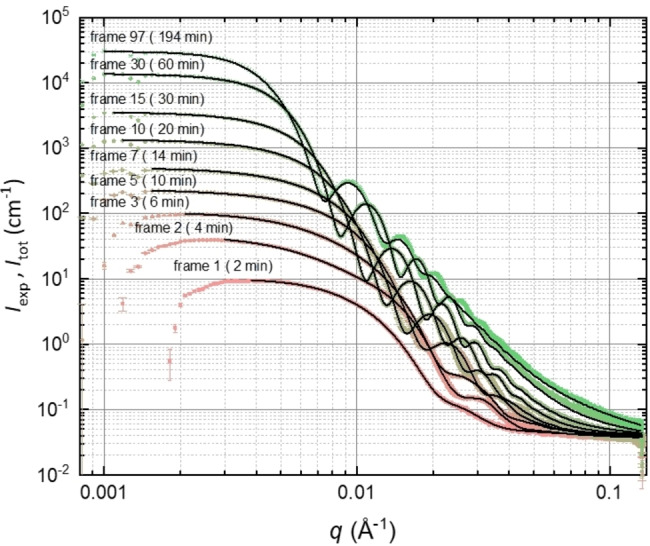
Selected SAXS patterns (*I*_exp_, colored symbols), recorded in situ during the PISA synthesis of PSMA_31_–PBzMA_2000_ spheres at 90 °C in mineral oil when targeting 10 % w/w solids. The corresponding fits (*I*_tot_, solid black lines) were obtained using the new SAXS models developed in this work. The SAXS patterns are plotted on an absolute intensity scale. The labels above each pattern indicate the respective frame number.

**Figure 3:**
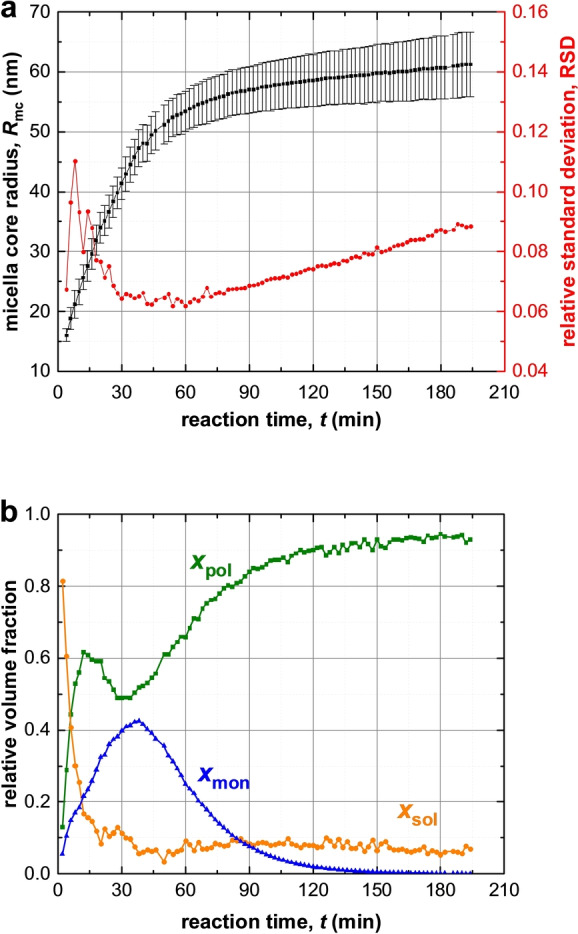
Time‐dependent evolution of various structural parameters determined by TR‐SAXS analysis during the PISA synthesis of PSMA_31_–PBzMA_2000_ nanoparticles in mineral oil at 90 °C. (a) Spherical micelle core radius *R*_mc_ (black squares; error bars correspond to the standard deviation in *R*_mc_) and its associated dispersity expressed via RSD (red circles). (b) Volume fraction of PBzMA chains (*x*_pol_, green squares), BzMA monomer (*x*_mon_, blue triangles) and mineral oil (*x*_sol_, orange circles) within the micelle cores.

Indeed, eq 1 produced a good fit to the SAXS pattern recorded for the final product (see Figure 2, uppermost pattern), indicating a final spherical micelle core radius of *R*_mc_=61.2 nm and 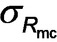
=5.4 nm. The relative standard deviation of the particle size [RSD, which is defined as the coefficient of variability ( 
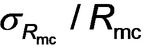
)] is 0.088, which suggests a relatively narrow size distribution (Figure 3a). This SAXS data fit also indicates minimal solvent plasticization of the spherical micelle cores by the mineral oil (*x*_sol_=0.07) (Figure 3a), which is consistent with NMR data obtained in earlier study.^[13c]^ The radius of gyration of the corona block of 1.6 nm indicated by SAXS analysis is consistent with calculations based on a Gaussian chain model.^[24]^ More specifically, the mean contour length of a PSMA_31_ stabilizer chain is estimated to be 7.9 nm (assuming that the length of two C−C bonds in an all *trans* configuration is 0.255 nm, hence 31 × 0.255 nm=7.9 nm). Given that the mean Kuhn length of the PSMA_31_ stabilizer is estimated to be around 1.53 nm (based on data available for PMMA^[25]^), an unperturbed radius of gyration for PSMA_31_ is calculated to be (7.9 nm× 1.53 nm/6)^0.5^=1.42 nm. The parameters obtained from the data fit for the final SAXS pattern were then used as initial parameters for fitting the penultimate SAXS pattern and this protocol was repeated for each subsequent pattern [N.B. The SAXS pattern number, *t_m_*, is directly related to the corresponding reaction time expressed in minutes according to the relation *m*=*t_m_*/2]. In principle, this data‐fitting strategy should minimize any uncertainty in each fitted parameter because this is constrained by results obtained for adjacent frames.

The total scattering intensity equation (eq 1) produced reasonably good fits to most of the SAXS patterns collected during the time‐resolved experiment, starting from the last frame (frame 97, *t*_97_=194 min) down to frame 7 inclusive (Figure 2). Since eq 1 only relates to **Stages 4** and **5** of the PISA synthesis, this suggests that no free copolymer chains were detected in the continuous phase after the first 12 min of the polymerization. However, the spherical micelle model did not provide satisfactory data fits for the first six frames even when accounting for a population of free copolymer chains (see **Stage 3** in Figure 1 and eq S2).

Close inspection of the SAXS data reveals that the first recorded SAXS pattern contains the first minimum of the particle form factor function, which is located at a slightly lower *q* value than that for the second pattern (0.020 Å^−1^ versus 0.023 Å^−1^, respectively), see Figures 2 and S3. This suggests that the initial nascent particles formed within the first 2 min are *larger* than those observed at a later stage. In addition, the scattering intensity for the second and third patterns has a non‐zero gradient in the Guinier region (0.0035 Å^−1^<*q*<0.015 Å^−1^), see Figures 2 and S3. In principle, this suggests the formation of weakly anisotropic nascent particles. This hypothesis is supported by analysis using a pair‐distance distribution function (PDDF) (Figure S4a), indicating the possible presence of particle dimers (Figure S4a, pattern corresponding to frame 2).^[26]^ It is conceivable that the initial micelles might immediately become elongated^[27]^ (or undergo fission to form two smaller micelles) before regaining their isotropic character at a later stage (see pattern 7 in Figures 2 and S3). However, analysis of the first six SAXS patterns confirmed that neither an ellipsoidal micelle model nor a spherical micelle dimer model provided satisfactory data fits. Since the intermediate species are observed in the presence of a relatively high concentration of unreacted BzMA monomer, an alternative explanation for the formation of relatively large particles within the first few minutes of the polymerization is that the growing copolymer chains could stabilize BzMA‐rich droplets (see Figure S5). To examine this hypothesis, a suitable SAXS model was developed (equations S34 and S35). Reasonable fits to the first six SAXS patterns were obtained by combining a BzMA‐rich droplet population with the spherical micelle population (eq S34), observed at later reaction times (see Figures 2 and S3). It is perhaps worth emphasizing that analysis of the curves fitted to the experimental SAXS patterns (Figure S4b) produced similar pair‐distance distribution functions to those reconstructed from the experimental SAXS patterns (Figure S4a). This two‐population model (Figure S5) indicates that there is a significant reduction in the relative volume fraction of the BzMA‐rich droplets within a few minutes of their formation, with the spherical micelle population dominating thereafter (Figure S6). More specifically, the BzMA‐rich droplets increase up to a limiting core radius of around 28 nm, while the spherical micelle core radius increases monotonically up to the same value before surpassing it (Figure S7). At this time point, the mean aggregation numbers for these two populations are comparable (Figure S8). Although this structural model is physically reasonable, it is not clear whether these two populations are formed independently or if there is a gradual transformation of the BzMA‐rich droplets into the spherical micelles. In order to address this issue in more detail, further time‐resolved SAXS experiments would be required that focus on the initial reaction period. However, this is beyond the scope of the current study.

Although the copolymer morphology is ill‐defined for the first few minutes of this PISA synthesis, useful structural parameters related to the reaction kinetics can be extracted from TR‐SAXS measurements. Unfortunately, SAXS lacks sufficient sensitivity to detect the molecularly‐dissolved copolymer chains shown in **Stages 2** and **3** of the synthesis (Figure 1). Nevertheless, the SAXS analysis suggests that pre‐micellar self‐assembly occurs at a very early stage and is likely to involve the formation of BzMA‐rich droplets. Moreover, spherical micelles (Figure 1, **Stage 3**) are also formed within the first few minutes. This means that **Stage 2** (Figure 1) must be neglected for the SAXS analysis, which reduces the number of structural parameters by excluding the population of molecularly‐dissolved copolymer chains from the scattering model.

**Figure 4:**
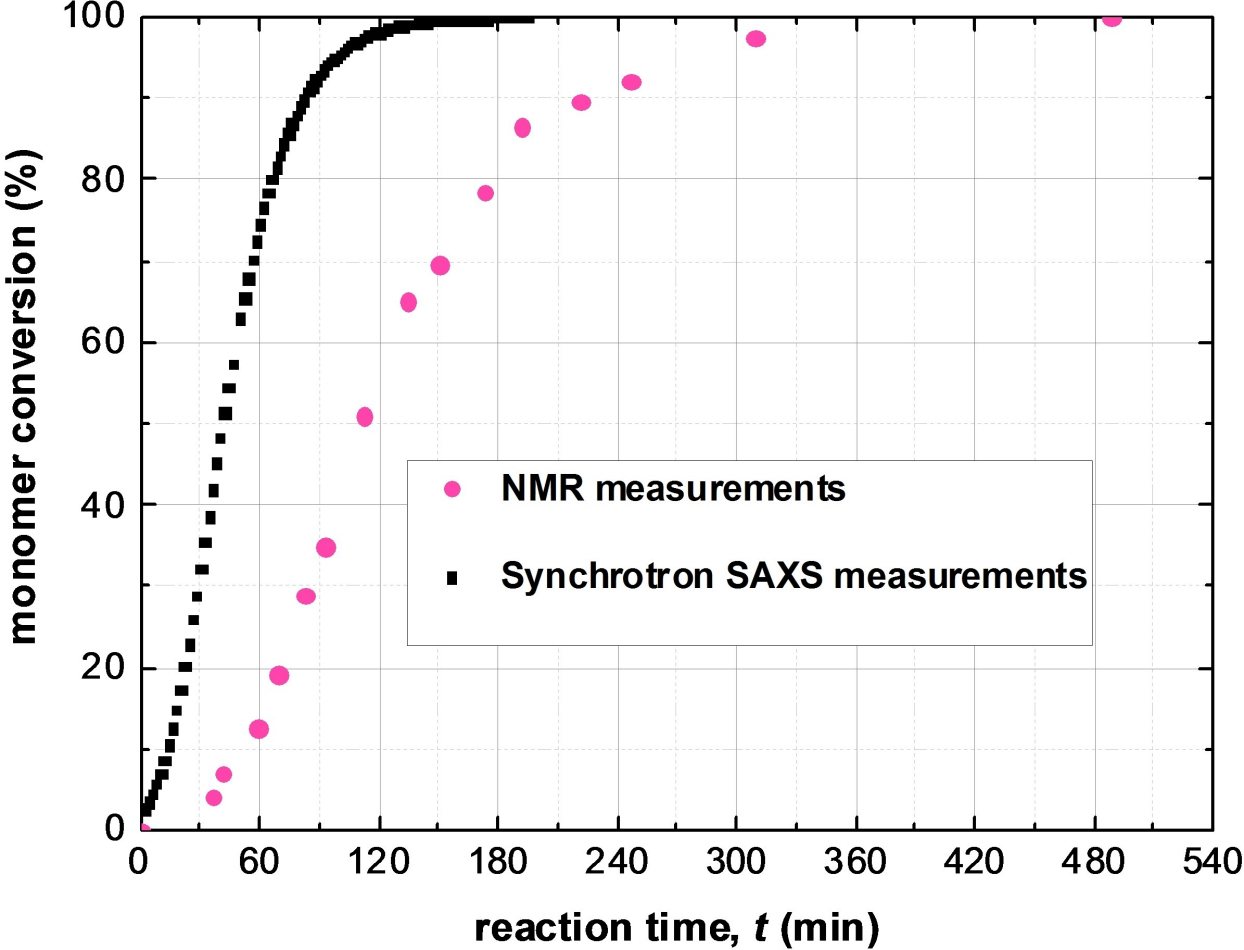
BzMA monomer conversion calculated from both synchrotron SAXS data (squares) and laboratory NMR results (circles) collected during PISA via RAFT synthesis of PSMA_31_–PBzMA_2000_ diblock copolymer at 90 °C in mineral oil at 10 % w/w solids.

In principle, the spherical micelle radius can be determined by DLS by periodic sampling of the reaction mixture.^[28]^ However, TR‐SAXS measurements provide much more detailed information (Figure 3b). For example, SAXS analysis indicates that the solvent volume fraction within the growing micelles (*x*_sol_) is initially high (>0.80) but drops to less than 0.10 within 20 min and is then further reduced to around 0.07 over the course of the BzMA polymerization. During the early stages of this reaction, the PBzMA chains are relatively short (DP<87, Figure 4) and hence remain molecularly dissolved in mineral oil. However, the growing PBzMA chains become increasingly solvophobic and micellar nucleation occurs once they reach a certain critical DP. The monomer volume fraction within the micelle cores (*x*_mon_) is initially low at the onset of micellar nucleation (Figure 3). However, as the BzMA polymerization progresses, further monomer diffuses into the micelles and displaces the mineral oil, which is no longer a good solvent for the growing PBzMA chains. Moreover, the rate of monomer mass transport is initially faster than the rate of polymerization, which leads to the gradual build‐up in the local monomer concentration (Figure 3b). The maximum value for *x*_mon_ is approximately 0.43, which is observed after around 38 min (Figure 3b) (or a BzMA conversion of 46 %, Figure 4). This relatively high BzMA concentration within the micelles significantly exceeds the BzMA concentration remaining in the continuous phase, which explains the pronounced rate acceleration. Moreover, this time point most likely corresponds to that for maximum chain mobility within the micelle cores—thereafter, the local concentration of BzMA monomer is reduced as the effective glass transition temperature increases for the growing PBzMA chains. The volume fraction of the growing PBzMA chains within the micelle cores (*x*_pol_) rapidly increases from 0.13 to 0.62 after micellar nucleation owing to the concomitant expulsion of solvent. However, a local maximum in *x*_pol_ is observed after 15 min (Figure 3b) owing to the gradual ingress of BzMA monomer into the micelle cores. *x*_pol_ then passes through a local minimum at around 30–32 min, with this time point lying close to that observed for the local maximum in *x*_mon_ (Figure 3b).

The monomer conversion vs. time curve derived from the TR‐SAXS measurements shows that almost all of the BzMA is converted into PBzMA within 120 min at 90 °C (Figure 4). This is much shorter than the 8 h timescale required for the equivalent laboratory‐scale synthesis (Figure 4). This discrepancy has been attributed to the high‐flux X‐ray synchrotron radiation producing an additional radical flux,^[29]^ most likely owing to its interaction with the mineral oil.^[13c]^ Previously, a single logistic function, which serves as a reasonable empirical approximation, was employed to renormalize the NMR‐derived kinetic data and hence calculate a conversion vs. time curve for the synchrotron SAXS measurements.[^13c^, ^30^]

**Figure 5:**
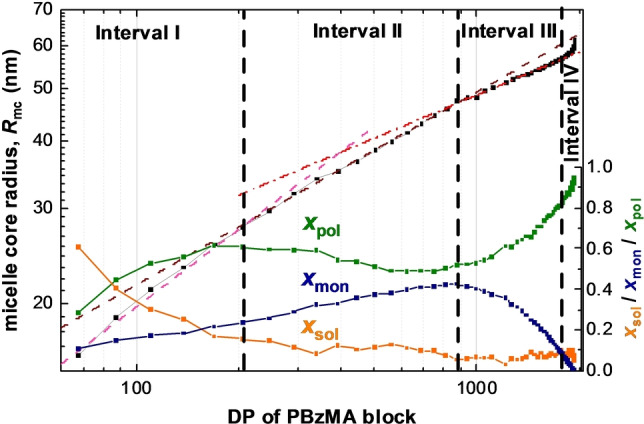
Double logarithmic plot of the mean micelle core radius *R*_mc_ (black squares) vs. PBzMA DP as determined by TR‐SAXS measurements performed during the PISA synthesis of PSMA_31_–PBzMA_2000_ spheres, plus the respective change in volume fraction of the BzMA monomer (*x*_mon_, blue triangles), the solvent (*x*_sol_, orange circles) and the PBzMA chains (*x*_pol_, green squares). Four distinct periods of micelle core radius growth are labeled as intervals I, II, III and VI (dashed vertical lines indicate each boundary). Power law fits to the micelle core radii for the first three intervals are indicated by colored dashed lines (pink for interval I, brown for interval II and red for interval III).

At first sight, the monotonic increase in micelle core radius after micellar nucleation (Figure 3a) appears to be straightforward and consistent with the earlier observations.^[13c]^ However, SAXS analysis reveals a much more complex scenario (Figures 3b and 4). Using a power law relationship (*R*_mc_=*k*_R_DP_⋅DP^*ω*^, where *k*_R_DP_ is the coefficient and *ω* is the exponent), the dependence of the micelle core radius, *R*_mc_, on the PBzMA DP shown in Figure 5 can be divided into four distinct time intervals. The first interval ranges from a PBzMA DP of around 70 (frame 2, 4 min) up to 200 (frame 7, 14 min) and follows a power law with an exponent *ω*_1_ of 0.48, Figure 5. The second interval is from 14 to 38 min, in which the limiting slope of *R*_mc_ as a function of DP approaches *ω*_2_=0.36 as DP tends to 1000 (i.e. frames 7–19), while the third interval is from 38 to 86 min, whereby the PBzMA DP increases from around 880 to 1780 (i.e. frames 19–43) and a satisfactory data fit was achieved using *ω*_3_=0.26 (Figure 5). The final interval is from 88 to 194 min (or frames 44–97), for which the PBzMA DP varies from around 1780 up to approximately 2000. No power law fit could be obtained in this case. This is most likely because the BzMA conversion is 99 % after 132 min (frame 66), so the PBzMA DP remains essentially unchanged (Figure 4).

**Figure 6:**
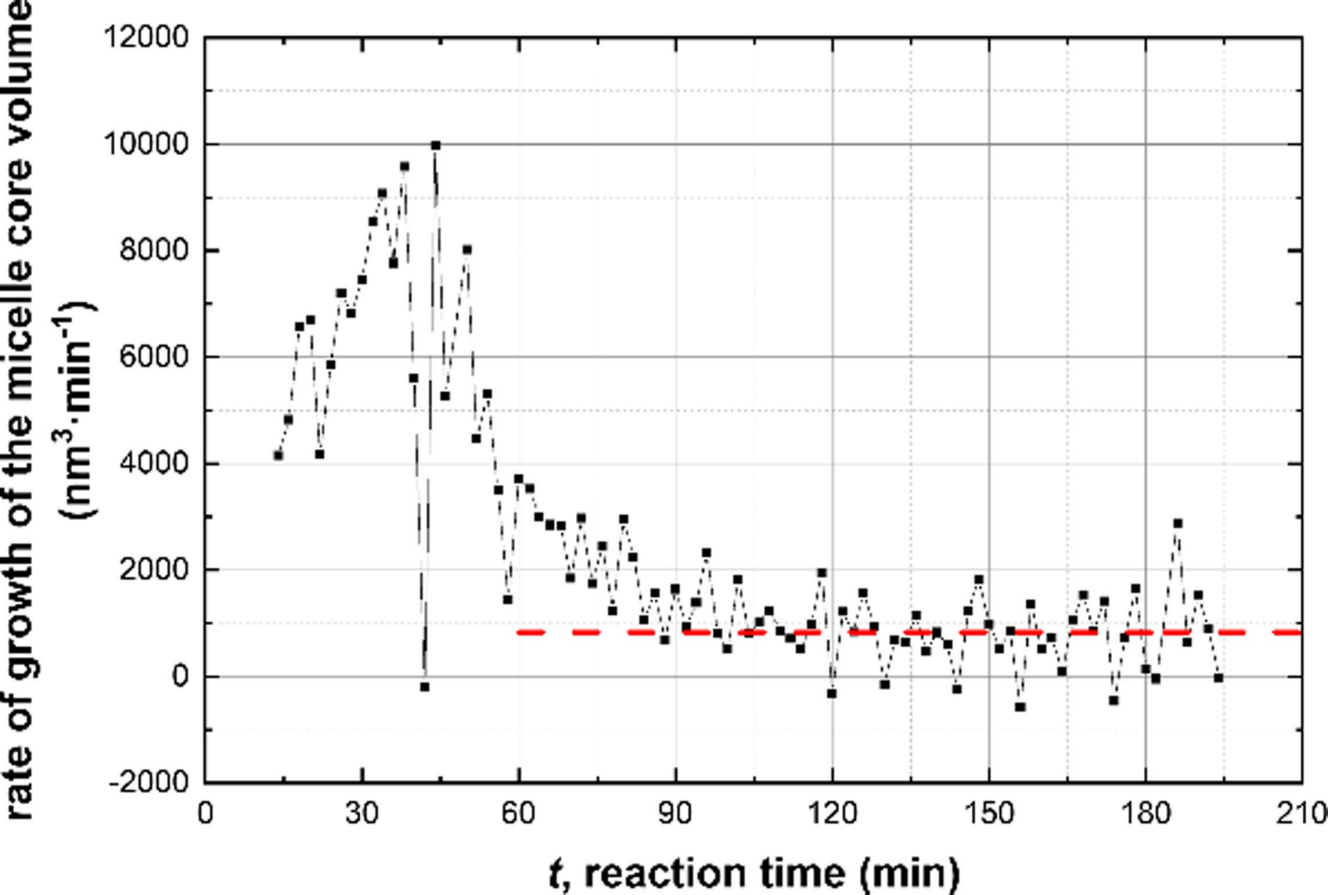
Time dependence of the rate of growth of the volume of the spherical micelle cores, [*R*_mc_^3^(t_m_)‐*R*_mc_^3^(*t*_m‐1_)]/(*t*_m_‐*t*_m‐1_), (black squares) during the PISA synthesis of PSMA_31_–PBzMA_2000_ nanoparticles at 90 °C in mineral oil at 10 % w/w solids. A constant linear function (dashed red line) provides a reference for possible PSMA_31_–PBzMA_2000_ nanoparticle relaxation at the end of the BzMA polymerization.

The initial power law scaling relationship for *R*_mc_ shows that the *ω*_1_ exponent lies close to 1/2. This suggests that the insoluble PBzMA chains remain partially stretched during interval I.[^13a^, ^13c^, ^31^] This is not unexpected, as this period coincides with a relatively high solvent volume fraction within the micelle cores (Figure 3). However, this solvent is gradually displaced from the micelle cores by the incoming BzMA monomer, which is a much better solvent/plasticizer for the growing PBzMA chains. During interval II, the increase in the micelle core radius is characterized by a smaller exponent, *ω*_2_, which is slightly higher than 1/3. Since the micelles are spherical, this suggests that the increase in their core volume is mainly because of the volume occupied by the PBzMA chains but with an additional contribution from a second component. Thus, to a reasonably good approximation, the increase in particle volume during this period simply involves uptake of BzMA monomer from the surrounding medium and its subsequent conversion into PBzMA chains (the so‐called ‘nanoreactor’ concept). The scaling exponent and *x*_mon_ are lower during interval III (Figure 5), which indicates a reduction in the rate of mass transport of BzMA monomer into the growing micelles. The fact that *ω*_3_ <1/3 is also expected: although monomer swelling leads to an increase in particle volume, concomitant conversion of monomer into polymer leads to densification and hence a reduction in the particle volume growth rate. In contrast to the first three intervals, no power law dependence on the PBzMA DP is observed during interval IV (Figure 5). The spherical micelle core radius over the entire experiment increases monotonically, while the RSD remains relatively low (Figure 3a). The latter parameter reaches its minimum value of about 0.06 within the first 45 min and then steadily increases up to around 0.09 by the end of the reaction. The increase in *R*_mc_ and the corresponding RSD observed after 88 min (Figure 3a), when the polymerization is close to completion (>90 % BzMA conversion, see Figure 4), may be the result of either micelle fusion and/or chain exchange mechanisms.[^21c^, ^32^] Indeed, SAXS analysis shows that the rate of growth of the micelle core volume, [*R*_mc_^3^(*t*_m_)‐*R*_mc_^3^(*t*_m‐1_)]/(*t*_m_ *‐t*_m‐1_), is approximately constant after 88 min (Figure 6). In principle, this suggests that an Ostwald ripening‐type mechanism might account for the change in particle size during interval IV.^[33]^ However, Ostwald ripening would require dissociation and diffusion of relatively long (and highly asymmetric) copolymer chains. Instead, it seems much more likely that the observed increase in size is the result of particle fusion. It should also be noted that the time point for maximum change in the particle volume (Figure 6) approximately corresponds to that for maximum monomer swelling (Figure 3b). This correlation indicates that the uptake and consumption of the BzMA monomer has a direct effect on the particle growth.

It is possible to relate the rate of polymerization, *R*_p_(*t*), to the instantaneous monomer concentration determined by SAXS. In principle, *R*_p_(*t*) can be written as^[34]^


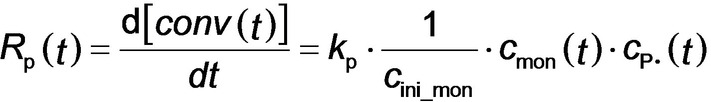




where *k*_p_ is the propagation rate coefficient, *c*_ini_mon_ is the initial molar concentration of monomer, *c*_mon_ is the molar concentration of monomer and *c*_P⋅_ is the molar concentration of polymer radicals. Moreover, eq 2 can be rewritten in terms of SAXS model parameters as:

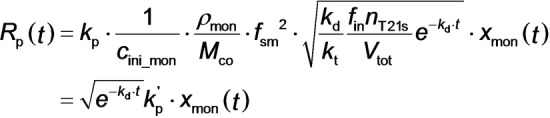




where *V*_tot_ is defined by eq S6, *M*_co_ is the molar mass of the repeat units within the solvophobic block (176 g mol^−1^ for BzMA), *k*_d_ is the initiator decomposition rate constant [calculated to be 1.92×10^−4^ s^−1^ from *k*_d_=ln(2)/*t*_1/2_ given that *t*_1/2_=1 h for T21s initiator at 90 °C^[35]^], *k*_t_ is the termination rate coefficient, *n*_T21s_ is the initial number of moles of T21s initiator, *f*_in_ is the initiator efficiency^[36]^ (often assumed to be less than unity and constant for T21s initiator),^[37]^ and *k*_p_′ is the composite propagation rate coefficient for the PISA synthesis (see equations S36–S42 for further details).

Accordingly, the overall rate of polymerization can be calculated from the TR‐SAXS data using:

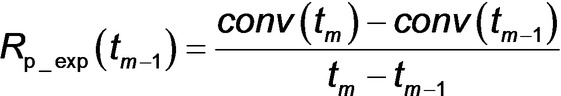




**Figure 7:**
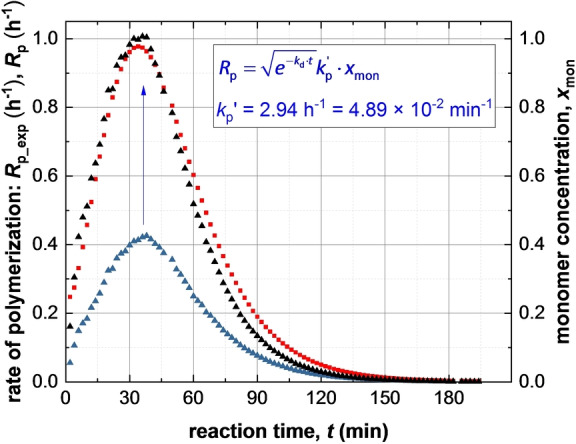
Time‐dependence of *x*_mon_ and *R*_p_exp_ obtained from SAXS results (blue triangles and red squares, respectively). The *x*_mon_ curve matches the *R*_p_exp_ curve if multiplied by 
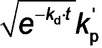
 (see black triangles representing 
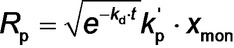
). For the sake of clarity, h^‐1^ unit was used for *R*_p_exp_ and *R*_p_.

Given that the rate of polymerization, *R*_p_exp_(*t*), calculated from SAXS analysis using eq 4 can be equated to *R*_p_(*t*) (eq 3), a least squares fit was applied using results obtained for all SAXS frames. A linear relationship between *R*_p_exp_(*t*) (eq 4) and *x*_mon_ (eq 3) was obtained, for which *k*_p_′=4.89×10^−2^ min^−1^ (Figure 7). This analysis requires further refinement because the synchrotron X‐ray beam significantly accelerates polymerization (Figure 4), which is attributed to the generation of a radical flux in addition to that provided by the initiator. Under such conditions, any reduction in initiator concentration may be neglected and *k*_d_ is assumed to be constant because the X‐ray irradiation provides a continuous supply of radicals. Indeed, this adjustment produced an even better correlation between *x*_mon_ and *R*_p_exp_ with a similar *k*_p_′ of 4.46×10^−2^ min^−1^ (Figure S9). Overall, these results confirm that the monomer concentration within the micelles (*x*_mon_) governs the overall rate of polymerization. In addition, using the calculated *k*_p_′ value, the typical *k*_t_ range of 10^6^–10^8^ L⋅mol^−1^⋅s^−1[37b]^ and assuming *f*_in_=1, *k*_p_ can be estimated from eq 3 as ranging from 7.09×10^2^ to 7.09×10^3^ L⋅mol^−1^⋅s^−1^. This range of values is consistent with the *k*_p_ value of 3.0×10^3^ L⋅mol^−1^⋅s^−1^ determined for the bulk polymerization of BzMA at 90 °C.^[38]^ This analysis suggests that TR‐SAXS should be a powerful *generic* technique for determining kinetic data during RAFT dispersion polymerization (and perhaps *any* dispersion polymerization formulation). However, it remains to be seen whether the established linear relationship is also applicable for RAFT aqueous *emulsion* polymerization formulations, for which the vast majority of the monomer is immiscible with the aqueous continuous phase.

Given that *x*_mon_ is the only time‐dependent parameter in eq 3, the relative volume fraction of BzMA monomer within the micelle cores dictates the rate of polymerization. However, *x*_mon_ not only depends on the rate of monomer supply according to eq S18 but also on *x*_sol_ and/or *x*_pol_. This suggests that invoking a suitable model to describe the expulsion of solvent from the micelle cores (Figure 5) could be employed to predict the change in *x*_sol_ over time.

Combining *R*_mc_ with *x*_mon_, *x*_sol_ and *x*_pol_ can be used to characterize micellar self‐assembly. In particular, the mean aggregation number (*N*_agg_, see eq S30), the average number of copolymer chains per unit surface area of micelle (*S*_agg_) and the average inter‐separation distance between adjacent copolymer chains packed at the surface of the micelle cores (*d*_int_) can be expressed using the following equation:

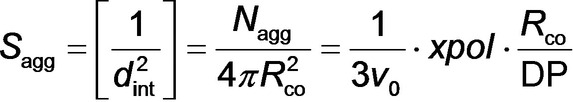




**Figure 8:**
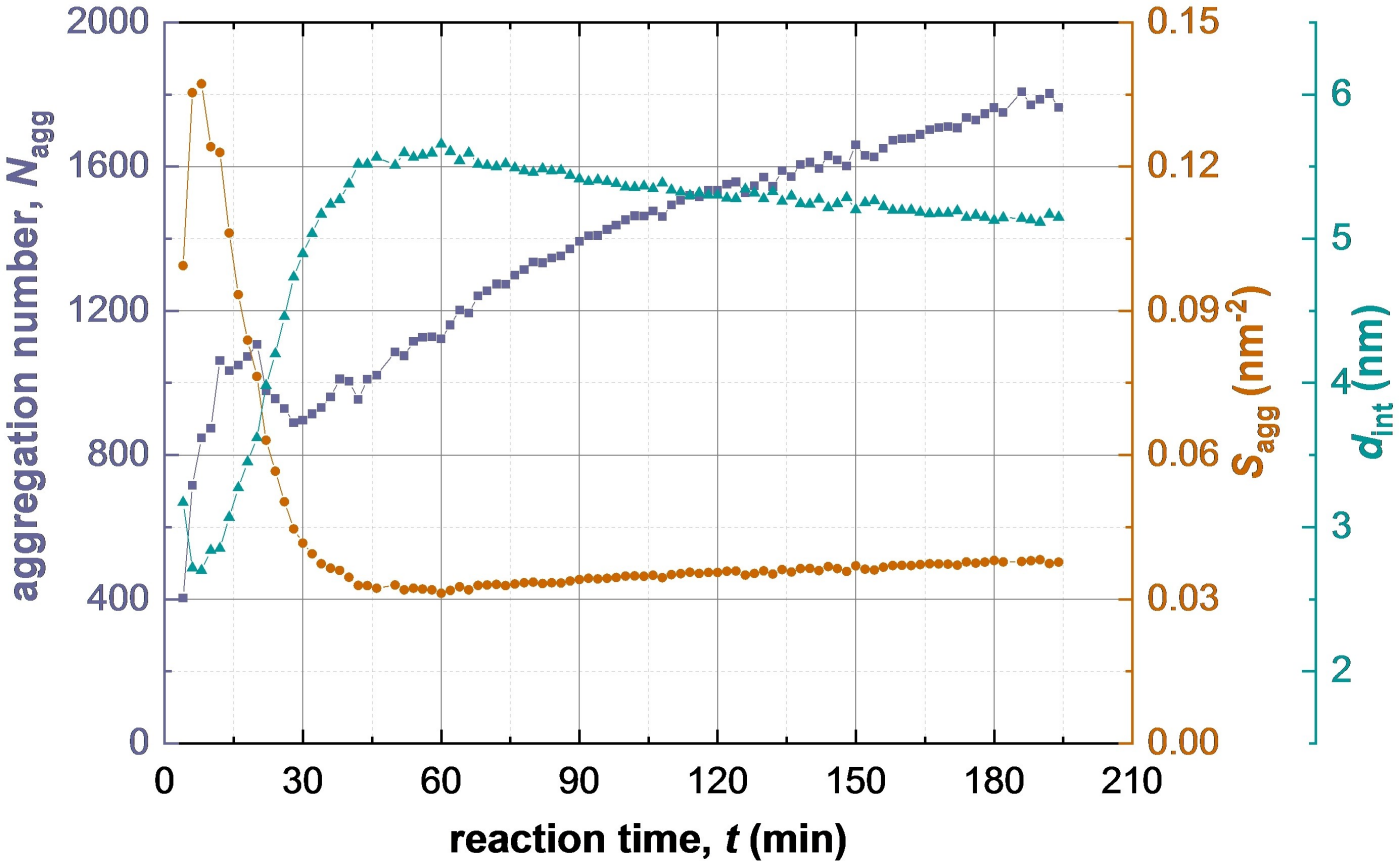
Evolution in the mean copolymer aggregation number (*N*_agg_) (purple squares), the mean number of copolymer chains per unit surface area of the micelles (*S*_agg_) (brown circles), and the mean distance between neighbouring copolymer chains within the micelles (*d*_int_) (green diamonds) during PISA, as determined by TR‐SAXS experiments.

Clearly, the micelle core radius must increase as the solvophobic PBzMA chains grow longer, even if there is no change in *N*_agg_. In practice, *N*_agg_ also increases during the BzMA polymerization (Figure 8, first 20 min). Jones et al.^[11]^ reported similar results for the RAFT dispersion polymerization of BzMA in ethanol. This results in a relatively high packing density for the copolymer chains: the radius of gyration of the PSMA_31_ stabilizer chains is around 1.6 nm, so *d*_int_ would be expected to be at least 3.2 nm to avoid any overlap with neighboring chains. However, the *d*_int_ gradually increases (accordingly, the copolymer packing density is gradually reduced) and attains an equilibrium value at around 60 min. After the initial sharp increase, a slight reduction in *N*_agg_ is observed after 30 min of the reaction. This coincides with the second interval for growth of the micelle cores (Figure 5). During this period, there is a power law relationship between *R*_mc_ and DP with an exponent of approximately 1/3. This means that *N*_agg_ mainly depends on *x*_pol_ but is not sensitive to *R*_mc_ during the second period (see eq S30). Thus, the reduction in *N*_agg_ should be attributed to relatively fast monomer diffusion into the micelle cores (Figure 3b). This is likely to release a minor fraction of copolymer chains into the continuous phase, which would then undergo self‐assembly to form new micelles. As the BzMA monomer is consumed, the volume fraction of PBzMA chains within the micelles increases. The steady growth in *N*_agg_ observed after 60 min (Figure 8) occurs when the polymerization is close to completion (Figure 4) and is likely to be related to relaxation via a micelle fusion mechanism (Figure 3a).

## Conclusion

A new structural model has been developed to describe the X‐ray scattering during RAFT dispersion polymerization, which is an important example of PISA. More specifically, combining the law of conservation of mass with an appropriate scattering model enables SAXS data acquired during the PISA synthesis of PSMA_31_–PBzMA_2000_ spherical nanoparticles in mineral oil at 90 °C to be analyzed in unprecedented detail. Importantly, this new approach enables the instantaneous monomer conversion to be directly determined. Moreover, the evolution in the mean particle radius, *R*_mc_, and the relative volume fractions of both unreacted monomer and solvent within the growing spherical micelle cores can be calculated, which provides a direct measure of the extent of monomer swelling and solvent plasticization of the growing nanoparticles during PISA. When combined with the volume fractions of mineral oil and BzMA monomer within the continuous phase (as calculated from the X‐ray scattering length density), these data enable the kinetics of this RAFT dispersion polymerization to be monitored. Furthermore, monomer ingress into (and concomitant solvent depletion from) the micelle cores can be monitored in situ by SAXS. This provides vital information that is simply not accessible using other characterization techniques. For example, the instantaneous rate of BzMA polymerization observed after micellar nucleation is proportional to the local BzMA concentration within the nanoparticle cores.

The synthesis of PSMA_31_–PBzMA_2000_ spherical nanoparticles comprises three steps. The initially soluble diblock copolymer chains first form relatively large ill‐defined nascent nuclei (step 1). These species are then transformed into a single population of well‐defined spherical micelles by either expelling some copolymer chains and/or undergoing fission (step 2). This is followed by micelle growth (step 3). A power law scaling relationship between the mean micelle core radius and the mean PBzMA DP indicates that step 3 can be subdivided into three distinct time intervals. For the first interval, which occurs immediately after micellar nucleation, the solvophobic PBzMA chains remain partially stretched (plasticized) within the micelle cores. During the second interval, the solvent is gradually displaced from the micelle cores by the incoming BzMA monomer. There is a monotonic increase in the local monomer concentration within the micelles up to a volume fraction of 0.43, which corresponds to the maximum rate of polymerization. Moreover, the rate of polymerization and the rate of monomer diffusion from the continuous phase into the growing micelle cores both remain constant, until a slower rate is eventually observed under monomer‐starved conditions. The third interval coincides with a reduction in the rate of monomer diffusion into the growing particles as this reagent becomes depleted within the continuous phase. Conversion of monomer into polymer under such monomer‐starved conditions necessarily leads to densification and hence a reduction in the rate of growth of the mean particle volume.

In the future, it would be useful to extend this new TR‐SAXS approach to monitor the evolution in copolymer morphology from spheres to worms to vesicles by employing a relatively short steric stabilizer block. Given that the strategy for SAXS data analysis developed in this work is based on the general principle of mass balance, it should be also applicable to other heterogeneous formulations involving the spatial segregation of reagents and products.

## Supporting Information

SAXS models, SAXS data for initial stages of the PISA synthesis, calculations of the polymerization rate and experimental methods used in this work can be found in the Supporting Information. The authors have cited additional references within the Supporting Information.^[39]^

## Conflict of interest

The authors declare no conflict of interest.

1

## Supporting information

As a service to our authors and readers, this journal provides supporting information supplied by the authors. Such materials are peer reviewed and may be re‐organized for online delivery, but are not copy‐edited or typeset. Technical support issues arising from supporting information (other than missing files) should be addressed to the authors.

Supporting Information

## Data Availability

The data that support the findings of this study are available in the Supporting Information of this article.
