# Supplementary material for: Determination of Reaction Kinetics by Time‐Resolved Small‐Angle X‐ray Scattering during Polymerization‐Induced Self‐Assembly: Direct Evidence for Monomer‐Swollen Nanoparticles
Source: Angew Chem Int Ed Engl. 2023 Dec 6;63(2):e202312119. doi: 10.1002/anie.202312119 (PMC10952692; doi:10.1002/anie.202312119)
Supplement: Supplementary file 1 — Supporting Information [file ANIE-63-0-s001.pdf]

Angewandte Chemie
Eine Zeitschrift der Gesellschaft Deutscher Chemiker

Supporting Information

Determination of Reaction Kinetics by Time-Resolved Small-Angle X-ray Scattering during Polymerization-Induced Self-Assembly: Direct Evidence for Monomer-Swollen Nanoparticles

G. Liao, M. J. Derry, A. J. Smith, S. P. Armes, O. O. Mykhaylyk**

Supporting Information for

Determination of Reaction Kinetics by Time-Resolved Small-angle X-ray Scattering during Polymerization-Induced Self-Assembly: Direct Evidence for Monomer-Swollen Nanoparticles

Guoxing Liao[1,2], Matthew J. Derry[1,3], Andrew J. Smith[4], Steven P. Armes[1,*] and Oleksandr O. Mykhaylyk[1,*]

[1]Dainton Building, Department of Chemistry, The University of Sheffield, Sheffield, S3 7HF, UK

[2]South China Advanced Institute for Soft Matter Science and Technology, School of Emergent Soft Matter, Guangdong Provincial Key Laboratory of Functional and Intelligent Hybrid Materials and Devices, South China University of Technology, Guangzhou, 510640, China

[3]Aston Advanced Materials Research Centre, Aston University, Aston Triangle, Birmingham, B4 7ET, UK

[4]Diamond House, Diamond Light Source Ltd, Didcot, OX11 0DE, UK.

Contents

Spherical micelle SAXS model 1

Solvent background scattering 8

SAXS model parameters used for analysis 9

Two-population micelle SAXS model 9

SAXS analysis of the initial stages of the PISA synthesis 11

Calculation of the rate of BzMA polymerization after micellar nucleation during the PISA synthesis of $PSMA_{31}$-$PBzMA_{2000}$ spherical nanoparticles at 10% w/w in mineral oil 14

Experimental Methods 16

References 18

Spherical micelle SAXS model

Bearing in mind both the polymerization and the *in situ* self-assembly that occurs during such RAFT-mediated PISA syntheses[1] (**Figure 1**), several simplifying assumptions can be made to assist derivation of a suitable structural model for TR-SAXS data analysis. First, the sealed capillary cell is a closed system. Thus, the law of conservation of mass can be invoked because there is essentially no change in the total mass of the reagents and the reaction products during the polymerization. Second, the reaction temperature and pressure are constant, so the density of each individual component does not change during PISA. Thus, the volume of each component can be calculated from its mass at any given reaction time, and the respective scattering length densities, which are related to the corresponding mass densities, can be considered to remain constant. This

implicitly assumes that both the mass density and the scattering length density of the diblock copolymer chains is independent of their state: they are either dissolved in solution or self-assembled in the form of spherical micelles. Third, all copolymer chains are assumed to have the same composition and the same degree of polymerization (in other words, the dispersity or M_w/M_n is assumed to be unity). Hence the instantaneous monomer conversion can be used to calculate the DP of the PBzMA block at any given time. Clearly, if the copolymer dispersity (M_w/M_n) is not unity, this affects the number of copolymer chains per micelle (or mean aggregation number). However, if the micelle size dispersity is relatively low, each micelle must contain a very similar number of copolymer chains. Furthermore, a low micelle size dispersity ensures that the relative amount of each of the four components present in the micelles (i.e. BzMA, solvent, PSMA and the growing PBzMA chains, which are related via mass balance), must be very similar regardless of the copolymer dispersity. This is because each micelle has essentially the same volume, so the mean overall reaction rate is equivalent to the reaction rate within each individual micelle). Fourth assumption to be made is that all spherical micelles have the same chemical composition and hence contain the same volume fractions of PBzMA, BzMA monomer and solvent within their cores. Fifth, all elemental volumes have the same composition at any given time and are equally representative of the whole system: this requirement is readily achievable for a homogeneous reaction mixture.[2] Finally, the mean aggregation number (or the average number of diblock copolymer chains per micelle) can vary so the micelle size distribution must have a finite, non-zero, width.

In general, a scattering signal recorded at any given intermediate monomer conversion during the polymerization should be composed of scattering from the solvent and monomer molecules, the molecularly dissolved diblock copolymer chains[3] and/or the spherical diblock copolymer micelles.[4] Furthermore, this scattering signal will be affected by interactions between the various components within the system. Thus, a suitable scattering equation for this system should comprise two sets of terms: one set associated with the shape and the internal structure of the scattering objects and a second set describing their interactions with each other. For the particular PISA formulation under investigation, the volume fraction of the soluble $PSMA_{31}$ precursor in the initial reaction mixture is around 0.02. By the end of **Stage 3** (**Figure 1**), it is likely that the volume fractions of both the diblock copolymer chains and the micelles are relatively low. Therefore, any interactions between such scattering objects can be neglected. For **Stages 1-3** (**Figure 1**), it follows that the general equation for the total scattering intensity (differential scattering cross-section per unit sample volume) can be expressed as a combination of scattering terms from *m* types of individual objects:

$$I_{\mathrm{tot}}(q)=\sum_{i=1}^{m}\phi_i I_i(q)+\left(1-\sum_{i=1}^{m}\phi_i\right)I_{\mathrm{bg}}=\sum_{i=1}^{m}\phi_i\frac{\int_0^\infty \Psi_i(\Upsilon_i)F_i(q,\Upsilon_i)S_i(q,\Upsilon_i)d^{n_i}\Upsilon_i}{\int_0^\infty \Psi_i(\Upsilon_i)V_i(\Upsilon_i)d^{n_i}\Upsilon_i}+\left(1-\sum_{i=1}^{m}\phi_i\right)I_{\mathrm{bg}} \quad \text{(S1)}$$

,

where *q* is the scattering vector length ($q = 4\pi\sin\theta/\lambda$, θ is one-half of the scattering angle and λ is the radiation wavelength; only the scalar form of this equation is considered); $I_i(q)$, ϕ_i and $\frac{\phi_i}{\int_0^\infty \Psi_i(\Upsilon_i)V_i(\Upsilon_i)d^{n_i}\Upsilon}$ are the scattering intensity, volume fraction and number density of the ith type of the individual objects, respectively; I_{bg} is the solvent background scattering, which is independent of *q*; $\Psi_i(\Upsilon_i)$, $F_i(q, \Upsilon_i)$ and $S_i(q, \Upsilon_i)$ are the multivariate normalized distribution function

$\left(\int_0^\infty \Psi_i(\Upsilon_i) d^{n_i}\Upsilon_i = 1 \right)$, the form factor (including volume and excess scattering length density of the components) and the structure factor of the ith type of the individual objects, respectively, and Υ_i represents n_i-tuple of structural parameters with finite, non-zero, dispersity describing i^{th} type of the individual objects. During **Stages 4** and **5** (**Figure 1**), most of the diblock copolymer chains are located within micelles and there are virtually no free copolymer chains present,[5] so the number of types of individual objects is reduced to unity (m = 1). In addition, as the DP of the insoluble block increases during the polymerization, this leads to a higher concentration of larger spherical micelles and hence inter-micelle interactions are no longer negligible. Thus, the scattering intensity (eq S1) for **Stages 4** and **5** must be modified as follows:

$$I_{\text{tot}}(q) = \phi_{\text{sm}} \frac{\int_0^\infty \Psi_{\text{sm}}(\Upsilon_{\text{sm}}) F_{\text{sm}}(q,\Upsilon_{\text{sm}}) S_{\text{sm}}(q,\Upsilon_{\text{sm}}) d^{n_{\text{sm}}}\Upsilon_{\text{sm}}}{\int_0^\infty \Psi_{\text{sm}}(\Upsilon_{\text{sm}}) V_{\text{sm}}(\Upsilon_{\text{sm}}) d^{n_{\text{sm}}}\Upsilon_{\text{sm}}} + (1-\phi_{\text{sm}}) I_{\text{bg}}, \tag{S2}$$

where the 'sm' subscript denotes terms corresponding to the spherical micelles, and $F_{\text{sm}}(q, \Upsilon_{\text{sm}})$ and $S_{\text{sm}}(q, \Upsilon_{\text{sm}})$ are functions describing the form factor and the structure factor of these micelles, respectively.[6] Since a single type of monomer (BzMA) and soluble precursor (PSMA_{31}) was employed for this PISA formulation, equations S1 and S2 can be rewritten for each stage as:

$$I_{\text{tot}}(q) = \phi_{\text{pc}} I_{\text{pc}}(q) + (1-\phi_{\text{pc}}) I_{\text{bg}}(q), \tag{S3}$$

for Stage 2

$$I_{\text{tot}}(q) = \phi_{\text{pc}} I_{\text{pc}}(q) + \phi_{\text{sm}} I_{\text{sm}}(q) + (1-\phi_{\text{pc}}-\phi_{\text{sm}}) I_{\text{bg}}(q), \tag{S4}$$

for Stage 3

$$I_{\text{tot}}(q) = \phi_{\text{sm}} I_{\text{sm}}(q) + (1-\phi_{\text{sm}}) I_{\text{bg}}(q), \tag{S5}$$

for Stages 4 and 5

where ϕ_{pc} is the volume fraction of polymer (diblock copolymer) chains in solution, and I_{pc} and I_{sm} are the scattering intensities arising from the soluble diblock copolymer chains and the spherical micelles respectively, representing the term with integrals in equations S1 and S2. Actually, the current study is mainly focused on events occurring in **Stages 4** and **5**. However, **Stages 1-3** are also considered here for the sake of completeness.

The total scattering intensity is proportional to the individual volume fractions of each component (equations S1-S5). In general, the total volume, V_{tot}, of the system can be expressed as:

$$V_{\text{tot}} = V_{\text{liq}} + V_{\text{pol}}, \tag{S6}$$

where V_{liq} and V_{pol} are the total volumes of the liquid and polymer components present in the system, respectively. Invoking the law of conservation of mass enables further relationships between the volume fractions to be deduced. Thus, the total volume of the diblock copolymers, V_{pol}, at any given reaction time can be expressed as:

$$V_{\text{pol}} = V_{\text{co}} + V_{\text{br}} = conv \cdot \frac{m_{\text{ini\_mon}}}{\rho_{\text{co}}} + \frac{m_{\text{br}}}{\rho_{\text{br}}}, \tag{S7}$$

where V_{co} is the total volume of the solvophobic blocks (in this study, PBzMA), as calculated from the instantaneous monomer conversion (*conv*), the initial monomer mass (m_{ini_mon}) and the mass density of the insoluble blocks (ρ_{co}), and V_{br} is the (constant) total volume of the soluble blocks as calculated from their mass (m_{br}) and mass density (ρ_{br}).

Given that just one monomer and a mixture of solvents are used for the polymerization and their ideal mixing takes place, the total volume of liquid present in the system can be expressed as:

$$V_{liq} = V_{mon} + V_{sol} = V_{mon} + \sum_{j=1}^{n_{sol}} V_{solj} = V_{mon} + \sum_{j=1}^{n_{sol}} \frac{m_{solj}}{\rho_{solj}}, \qquad \text{(S8)}$$

where V_{mon} is the volume of unreacted monomer at any given time, V_{sol} is the solvent volume (which is composed, in general, of n_{sol} components), and V_{solj} is the volume of the jth solvent component as defined by its mass, m_{solj}, divided by its mass density, ρ_{solj}, respectively. V_{mon} in eq S8 can be defined as:

$$V_{mon} = (1 - conv) \cdot \frac{m_{ini\_mon}}{\rho_{mon}}, \qquad \text{(S9)}$$

where ρ_{mon} is the mass density of the monomer (e.g. BzMA).

During the polymerization, the unreacted monomer becomes preferentially located within the micelle cores.[2, 7] Moreover, the solvent can plasticize the micelle cores.[8] Thus the liquid components can be subdivided into two parts, with one part being located within the micelle cores while the other remains within the continuous phase. Therefore, the total volume of solvent located within the micelle cores (V_{in_sol}) and in the external reaction solution (V_{ex_sol}) can be expressed as:

$$\begin{cases} V_{in\_sol} = \sum_{j=1}^{n_{sol}} f_{solj} V_{solj} \\ V_{ex\_sol} = \sum_{j=1}^{n_{sol}} \left(1 - f_{solj}\right) V_{solj} \end{cases}, \qquad \text{(S10)}$$

where f_{solj} is the volume fraction of jth solvent located within the micelle cores. By analogy, the total volume of monomer located within the micelles and the continuous phase (V_{in_mon} and V_{ex_mon}, respectively) can be expressed as:

$$\begin{cases} V_{in\_mon} = f_{mon} V_{mon} \\ V_{ex\_mon} = \left(1 - f_{mon}\right) V_{mon} \end{cases}, \qquad \text{(S11)}$$

where f_{mon} is the volume fraction of monomer within the micelle cores. According to equations S10 and S11, the total volume of liquid components within the micelle cores can be written as:

$$V_{in\_liq} = V_{in\_sol} + V_{in\_mon} = \sum_{j=1}^{n_{sol}} f_{solj} V_{solj} + f_{mon} V_{mon}. \qquad \text{(S12)}$$

Accordingly, the total volume of external liquid can be written as:

$$V_{ex\_liq} = V_{ex\_mon} + V_{ex\_sol}. \qquad \text{(S13)}$$

In principle, diblock copolymer chains can be either molecularly dissolved in the solvent and/or undergo self-assembly to form micelles (see **Figures 1** and **2** and equations S3-S5). Thus, in general the total amount of copolymer chains should be subdivided into two components:

$$1 = f_{pc} + f_{sm}, \tag{S14}$$

where f_{pc} is the fraction of free copolymer chains remaining in solution and f_{sm} is the fraction of copolymer chains that form the spherical micelles. Thus, the component volume fractions for equations S3-S5 can be expressed as:

$$\phi_{pc} = \frac{f_{pc} V_{pol}}{V_{tot}} \tag{S15}$$

$$\phi_{sm} = \frac{(1 - f_{pc}) V_{pol} + V_{in\_liq}}{V_{tot}}, \tag{S16}$$

where f_{pc} = 1 for **Stage 2** (eq 3) and f_{pc} = 0 for **Stages 4** and **5** (eq S5), see Figure 1. Hence, the volume fraction of external liquid responsible for background scattering in equations S3-S5 can be expressed as:

$$\phi_{ex} = 1 - \phi_{pc} - \phi_{sm} = \frac{V_{ex\_liq}}{V_{tot}}. \tag{S17}$$

In addition, the component volumes defined by equations S7, S10 and S11 enable volume fractions of monomer (e.g. BzMA), solvophobic block (e.g. PBzMA) and solvent (e.g. mineral oil) localized within the micelle cores to be expressed:

$$\begin{cases} x_{mon} = \dfrac{V_{in\_mon}}{f_{sm} V_{co} + V_{in\_mon} + V_{in\_sol}} \\ x_{pol} = \dfrac{f_{sm} V_{co}}{f_{sm} V_{co} + V_{in\_mon} + V_{in\_sol}} \\ x_{sol} = \dfrac{V_{in\_sol}}{f_{sm} V_{co} + V_{in\_mon} + V_{in\_sol}} \end{cases}. \tag{S18}$$

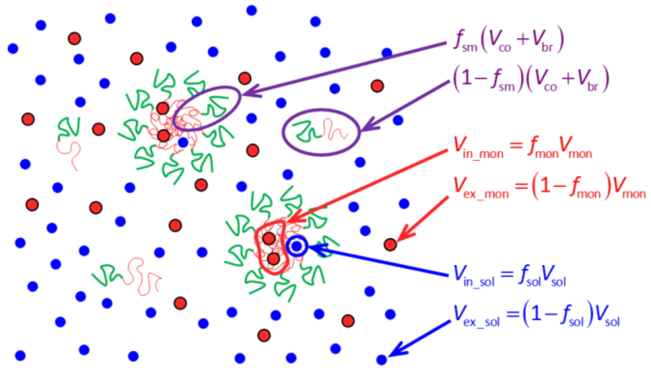

Figure S1. Schematic representation of the distribution of individual components for a PISA synthesis based on a dispersion polymerization formulation. f_{sol}, f_{mon} and f_{sm} are the volume fractions of solvent (blue spheres), monomer (red spheres), and diblock copolymer chains (curved green and red lines), which are located within the spherical micelles. V_{mon} is the volume of unreacted monomer at any given time, V_{sol} is the solvent volume, V_{co} is the total volume of the solvophobic blocks and V_{br} is the total volume of the soluble blocks.

Following prior work on the analysis of form and structure factors for spherical micelles,[6, 9] Υ_{sm} n_{sm}-tuple can be replaced with a single parameter by assuming that the micelle core radius (r_{mc}) is the only parameter that possesses a finite dispersity. Accordingly, the X-ray scattering intensity for the spherical micelles described by equations S2 and S5 can be expressed as:

$$I_{sm}(q)=\frac{\int_0^\infty \Psi_{sm}(r_{mc})\left(F_{sm}(q,r_{mc})+\int_0^\infty \Psi_{sm}(r'_{mc})A_{sm}(q,r_{mc})A_{sm}(q,r'_{mc})\left[S(q,r_{mc},r'_{mc})-1\right]dr'_{mc}\right)dr_{mc}}{\int_0^\infty \Psi_{sm}(r_{mc})V_{sm}(r_{mc})dr_{mc}}. \quad \text{(S19)}$$

The micelle form factor in eq S19 is defined as:

$$F_{sm}(q,r_{mc})=n_{agg}^2\beta_{mc}^2A_{mc}^2(q,r_{mc})+n_{agg}(n_{agg}-1)\beta_{br}^2\left[A_{br}(q,R_{g\_br})\frac{\sin q(r_{mc}+R_{g\_br})}{q(r_{mc}+R_{g})}\right]^2$$
$$+n_{agg}\beta_{br}^2F_{br}(q,R_g)+2n_{agg}^2\beta_{mc}\beta_{br}A_{mc}(q,r_{mc})A_{br}(q,R_{g\_br})\frac{\sin q(r_{mc}+R_{g\_br})}{q(r_{mc}+R_{g\_br})}, \quad \text{(S20)}$$

Where, for the sake of simplicity, it is assumed that there is no penetration of the corona chains within the micelle cores.[6] The micelle core scattering amplitude in eq S20 is expressed as:

$$A_{mc}(q,r_{mc})=\frac{3(\sin qr_{mc}-qr_{mc}\cos qr_{mc})}{q^3r_{mc}^3}e^{-\frac{1}{2}q^2\sigma_t^2}, \quad \text{(S21)}$$

where the exponent term with a width σ_t describes a sigmoidal interface between the two blocks at the micelle corona surface. The numerical value for σ_t was fixed at 2.5 Å during fitting. The aggregation number (eq S20) is defined as:

$$n_{agg}=x_{pol}\cdot\frac{\frac{4}{3}\pi r_{mc}^3}{v_{co}}, \quad \text{(S22)}$$

where v_{co} is the volume of a single solvophobic block as calculated from $v_{co}=\frac{M_{co}}{\rho_{co}\cdot N_A}\cdot \mathrm{DP}$, where M_{co} is the molar mass of the repeat units within the solvophobic block, N_A is Avogadro's constant and DP is the mean degree of polymerization of the solvophobic block calculated using $\mathrm{DP}=\frac{m_{ini\_mon}}{M_{co}}\frac{M_{br}}{m_{br}}\cdot conv$, where M_{br} is the number-average molecular weight of the soluble precursor block as determined by ^{1}H NMR spectroscopy. Pay attention that DP is also used as an abbreviation (and not as a defined parameter) for the degree of polymerisation especially at the beginning of the paper. It is clear that n_{agg} may be affected by the diblock copolymer dispersity, which is assumed to be unity in the model. In practice, this parameter is always greater than unity, which changes the number of solvophilic stabilizer chains located in the micelle corona. However, these PSMA chains only scatter weakly compared to the PBzMA cores, so they make only a rather small contribution to the overall X-ray scattering intensity and, consequently, have an insignificant effect on the results of SAXS analysis.
The excess scattering length density for the micelle cores in eq S20 is defined as:

$$\beta_{mc}=\frac{v_{co}}{x_{pol}}(\xi_{mc}-\xi_{bg}), \quad \text{(S23)}$$

where the scattering length density of the micelle cores is calculated as the volume-average of the following components:

$$\xi_{\text{mc}} = \frac{\xi_{\text{mon}} f_{\text{mon}} V_{\text{mon}} + \sum_{j=1}^{n_{\text{sol}}} \xi_{\text{sol}j} f_{\text{sol}j} V_{\text{sol}j} + \xi_{\text{co}} f_{\text{sm}} V_{\text{co}}}{f_{\text{mon}} V_{\text{mon}} + \sum_{j=1}^{n_{\text{sol}}} f_{\text{sol}j} V_{\text{sol}j} + f_{\text{sm}} V_{\text{co}}}, \qquad \text{(S24)}$$

where ξ_{mon}, $\xi_{\text{sol}j}$ and ξ_{co} are the scattering length densities of the monomer, the jth solvent component and the solvophobic block, respectively. The background scattering length density in eq S23 can be calculated using:

$$\xi_{\text{bg}} = \frac{\xi_{\text{mon}}\left(1-f_{\text{mon}}\right)V_{\text{mon}} + \sum_{j=1}^{n_{\text{sol}}} \xi_{\text{sol}j}\left(1-f_{\text{sol}j}\right)V_{\text{sol}j} + \left(1-f_{\text{sm}}\right)\left(\xi_{\text{co}}V_{\text{co}} + \xi_{\text{br}}V_{\text{br}}\right)}{\left(1-f_{\text{mon}}\right)V_{\text{mon}} + \sum_{i=1}^{n_{\text{sol}}}\left(1-f_{\text{sol}j}\right)V_{\text{sol}j} + \left(1-f_{\text{sm}}\right)\left(V_{\text{co}} + V_{\text{br}}\right)}, \qquad \text{(S25)}$$

where ξ_{br} is the scattering length density of the steric stabilizer (e.g. the PSMA block), for which the excess scattering length density in eq S20 is defined as:

$$\beta_{\text{br}} = v_{\text{br}}\left(\xi_{\text{br}} - \xi_{\text{bg}}\right), \qquad \text{(S26)}$$

where the volume occupied by a single steric stabilizer chain is calculated using $v_{\text{br}} = \frac{M_{\text{br}}}{\rho_{\text{br}} \cdot N_{\text{A}}}$. The scattering amplitude of spherical micelles, $A_{\text{sm}}(q,r_{\text{mc}})$, in eq S20 is defined as:

$$A_{\text{sm}}\left(q,r_{\text{mc}}\right) = n_{\text{agg}} \cdot \left[\beta_{\text{mc}} A_{\text{mc}}\left(q,r_{\text{mc}}\right) + \beta_{\text{br}} A_{\text{br}}\left(q,R_{\text{g\_br}}\right) \frac{\sin q\left(r_{\text{mc}} + R_{\text{g\_br}}\right)}{q\left(r_{\text{mc}} + R_{\text{g\_br}}\right)}\right], \qquad \text{(S27)}$$

where the form factor amplitude for the steric stabilizer chains is defined as $A_{\text{br}}\left(q,R_{\text{g\_br}}\right) = \frac{1-\exp\left(-q^2 R_{\text{g\_br}}^2\right)}{q^2 R_{\text{g\_br}}^2}$,[6] and $R_{\text{g\_br}}$ is its radius of gyration. The self-correlation term for the steric stabilizer chains in eq S20 is described by the Debye function $F_{\text{br}}\left(q,R_{\text{g\_br}}\right) = \frac{2\left[\exp\left(-q^2 R_{\text{g\_br}}^2\right) - 1 + q^2 R_{\text{g\_br}}^2\right]}{q^4 R_{\text{g\_br}}^4}$.[10] The structure factor term in eq S19 corresponding to inter-micelle interactions can be described using the hard-sphere structure factor solved by the Percus-Yevick closure relation:[9]

$$S\left(q,r_{\text{mc}},r'_{\text{mc}}\right) = S_{\text{PY}}\left(q,R_{\text{sf}},V_{\text{sf}}\right), \qquad \text{(S28)}$$

where V_{sf} is the effective volume fraction of the interacting micelles and the mean inter-micelle distance is defined as $2R_{\text{sf}} = r_{\text{mc}} + r'_{\text{mc}} + 2\Delta R$, where $2\Delta R$ is the shortest distance between neighbouring micelle cores with respective core radii r_{mc} and r'_{mc}.

The dispersity of the micelle core radius is assumed to follow a Gaussian distribution. Thus, the normalized distribution function in eq S19 can be expressed as:

$$\Psi\left(r_{\text{mc}}\right)=\frac{\exp\left[-\frac{1}{2}\left(\frac{r_{\text{mc}}-R_{\text{mc}}}{\sigma_{R_{\text{mc}}}}\right)^2\right]}{\sqrt{2\pi}\sigma_{R_{\text{mc}}}}, \tag{S29}$$

where R_{mc} is the mean radius of the spherical micelle core and $\sigma_{R_{\text{mc}}}$ is its standard deviation. Thus, the mean aggregation number, N_{agg}, is defined as:

$$N_{\text{agg}}=x_{\text{pol}}\cdot\frac{V_{\text{mc-co}}}{v_{\text{co}}}=x_{\text{pol}}\cdot\frac{\frac{4}{3}\pi R_{\text{mc}}^3}{v_{\text{co}}}, \tag{S30}$$

where $V_{\text{mc-co}}$ is the mean volume of the spherical micelle cores.

Assuming that any particle of a given size is always surrounded by particles of the same size, a local monodisperse approximation can be used to simplify eq S19:[11]

$$I_{\text{sm}}(q)=\frac{\int_0^\infty \Psi_{\text{sm}}\left(r_{\text{mc}}\right)\left(F_{\text{sm}}\left(q,r_{\text{mc}}\right)+A_{\text{sm}}^2\left(q,r_{\text{mc}}\right)\left[S\left(q,r_{\text{mc}}\right)-1\right]\right)dr_{\text{mc}}}{\int_0^\infty \Psi_{\text{sm}}\left(r_{\text{mc}}\right)V_{\text{sm}}\left(r_{\text{mc}}\right)dr_{\text{mc}}}, \tag{S31}$$

where, like eq S28, the structure factor term is expressed as $S\left(q,r_{\text{mc}}\right)=S_{\text{PY}}\left(q,R_{\text{sf}},V_{\text{sf}}\right)$ and $R_{\text{sf}}=r_{\text{mc}}+\Delta R$. Fitting selected SAXS patterns recorded during PISA synthesis using eq S19 and eq S31 confirmed that these two approaches gave similar results. However, the latter approach (eq S31) was chosen for the analysis as it required a significantly shorter computing time for SAXS data fitting.

Solvent background scattering

The solvent background scattering term, I_{bg}, is included in the SAXS model (eq S1). To account for any change of I_{bg} over the reaction time, further SAXS patterns were recorded at 90 °C for mineral oil containing 2.0-10.0% w/w BzMA monomer (w_{mon}). This range of monomer concentrations encompasses the initial BzMA concentration of 8.0% w/w used for the PISA synthesis. Experimental solvent background scattering ($I_{\text{exp\_bg}}$), independent of q, has been obtained from the reduced SAXS patterns collected for the mineral oil and BzMA monomer mixtures (**Figure S2**). Empirically, the observed $I_{\text{exp\_bg}}$ dependence on the monomer concentration can be interpolated by a cubic polynomial:

$$I_{\text{bg}}=I_{\text{bg}}\left(w_{\text{mon}}\right)=A_1+A_2\cdot w_{\text{mon}}+A_3\cdot w_{\text{mon}}^2+A_4\cdot w_{\text{mon}}^3, \tag{S32}$$

where A_1, A_2, A_3 and A_4 are the fitting parameters and w_{mon} can be expressed as

$$w_{\text{mon}}=\frac{\left(1-f_{\text{mon}}\right)m_{\text{mon}}}{\left(1-f_{\text{mon}}\right)m_{\text{mon}}+\sum_{j=1}^{n_{\text{sol}}}\left(1-f_{\text{sol}j}\right)m_{\text{sol}j}}. \tag{S33}$$

Eq S32 provided a satisfactory match to the experimental data (**Figure S2**), thus this analytical expression was incorporated into the SAXS model (eq S1).

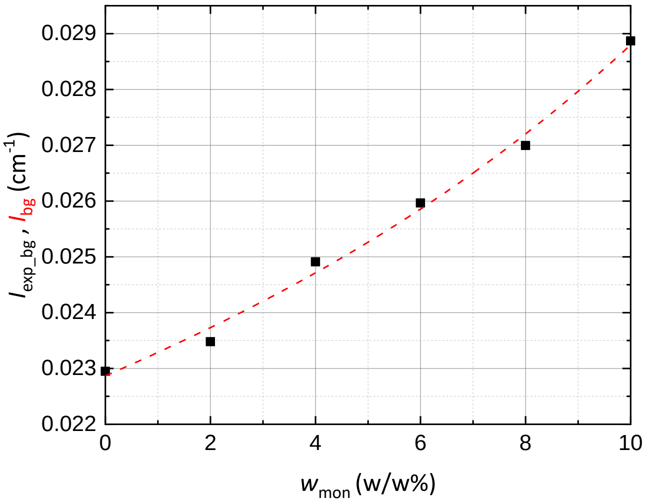

Figure S2. Variation in the X-ray scattering intensity (I_{exp_bg}) with BzMA monomer concentration in mineral oil (w_{mon} in w/w%). The experimental data (black squares) are fitted using the following cubic polynomial equation (eq S32; red dashed line): $I_{bg} = 0.0229 + 4.09 \times 10^{-4} w_{mon} + 9.40 \times 10^{-6} w_{mon}^2 + 8.95 \times 10^{-7} w_{mon}^3$.

SAXS model parameters used for analysis

Table S1. Summary of the independent model parameters used for SAXS data fitting.

Fitted parameters	Symbol
copolymer volume fraction assembled in micelles	ϕ_{sm}
copolymer volume fraction in the solution	ϕ_{pc}
mean core radius of copolymer micelles	R_{mc}
standard deviation of the micelle core radius	$\sigma_{R_{mc}}$
radius of gyration of hydrophilic (micelle corona) block	R_{g_br}
effective volume fraction of the interacting copolymer micelles	V_{sf}
half of the shortest distance between the micelle core interfaces	ΔR
volume fraction of monomer within the micelle cores	x_{mon}
volume fraction of solvent within the micelle cores	x_{sol}
instantaneous monomer conversion	*conv*
volume fraction of the BzMA-rich droplets	ϕ_{su}*
mean radius of the BzMA-rich droplets	R_{su}*
standard deviation of the BzMA-rich droplet radius	$\sigma_{R_{su}}$*
radius of gyration of the PBzMA block in the BzMA-rich droplets	R_{g_co}*

* Additional parameters used for SAXS data fitting by the two-population micelle model.

Two-population micelle SAXS model

The spherical micelle model is not applicable for the first six frames of the SAXS patterns. Instead, a two-population model that includes a population of spherical copolymer micelles and a

population of BzMA-rich droplets is introduced to describe the early stages of this PISA synthesis. Thus, eq 19 is rewritten as:

$$I_{\mathrm{tot}}(q)=\phi_{\mathrm{sm}}\frac{\int_0^\infty \Psi_{\mathrm{sm}}(r_{\mathrm{mc}})\left(F_{\mathrm{sm}}(q,r_{\mathrm{mc}})+\int_0^\infty \Psi_{\mathrm{sm}}(r'_{\mathrm{mc}})A_{\mathrm{sm}}(q,r_{\mathrm{mc}})A_{\mathrm{sm}}(q,r'_{\mathrm{mc}})\left[S(q,r_{\mathrm{mc}},r'_{\mathrm{mc}})-1\right]dr'_{\mathrm{mc}}\right)dr_{\mathrm{mc}}}{\int_0^\infty \Psi_{\mathrm{sm}}(r_{\mathrm{mc}})V_{\mathrm{sm}}(r_{\mathrm{mc}})dr_{\mathrm{mc}}}$$
$$+\phi_{\mathrm{su}}\frac{\int_0^\infty \Psi_{\mathrm{su}}(r_{\mathrm{su}})\left(F_{\mathrm{su}}(q,r_{\mathrm{su}})+\int_0^\infty \Psi_{\mathrm{su}}(r'_{\mathrm{su}})A_{\mathrm{su}}(q,r_{\mathrm{su}})A_{\mathrm{su}}(q,r'_{\mathrm{su}})\left[S(q,r_{\mathrm{su}},r'_{\mathrm{su}})-1\right]dr'_{\mathrm{su}}\right)dr_{\mathrm{su}}}{\int_0^\infty \Psi_{\mathrm{su}}(r_{\mathrm{su}})V_{\mathrm{su}}(r_{\mathrm{su}})dr_{\mathrm{su}}},\quad \text{(S34)}$$
$$+(1-\phi_{\mathrm{sm}}-\phi_{\mathrm{su}})I_{\mathrm{bg}}(w_{\mathrm{mon}})$$

where ϕ_{su} is the BzMA-rich droplet volume fraction, r_{su} is the BzMA-rich droplet core radius. It is assumed that the distribution function for the particle radius (Ψ_{su}) has a Gaussian distribution with a mean particle core radius of R_{su} and an associated standard deviation of $\sigma_{R_{\mathrm{su}}}$. $S(q,r_{\mathrm{mc}},r'_{\mathrm{mc}})$ and $S(q,r_{\mathrm{su}},r'_{\mathrm{su}})$ are hard sphere structure factors solved by the Percus-Yevick closure relation. Since the volume concentration of the scattering objects was relatively low at this early stage of the synthesis, their structure factors were taken to be unity [i.e., $S(q,r_{\mathrm{mc}},r'_{\mathrm{mc}})$ = $S(q,r_{\mathrm{su}},r'_{\mathrm{su}})$ = 1]. The form factor for the BzMA-rich droplet in eq S34 is defined as:

$$\begin{aligned}F_{\mathrm{su}}(q,r_{\mathrm{su}})&=\beta_{\mathrm{su}}^2A_{\mathrm{su\_s}}^2(q,r_{\mathrm{su}})\\&+n_{\mathrm{agg\_su}}\beta_{\mathrm{br}}^2F_{\mathrm{br}}(q,R_{\mathrm{g\_br}})+n_{\mathrm{agg\_su}}(n_{\mathrm{agg\_su}}-1)\beta_{\mathrm{br}}^2\left[A_{\mathrm{br}}(q,R_{\mathrm{g\_br}})\frac{\sin q(r_{\mathrm{su}}+R_{\mathrm{g\_br}})}{q(r_{\mathrm{su}}+R_{\mathrm{g\_br}})}\right]^2\\&+n_{\mathrm{agg\_su}}\beta_{\mathrm{co}}^2F_{\mathrm{co}}(q,R_{\mathrm{g\_co}})+n_{\mathrm{agg\_su}}(n_{\mathrm{agg\_su}}-1)\beta_{\mathrm{co}}^2\left[A_{\mathrm{co}}(q,R_{\mathrm{g\_co}})\frac{\sin q(r_{\mathrm{su}}-R_{\mathrm{g\_co}})}{q(r_{\mathrm{su}}-R_{\mathrm{g\_co}})}\right]^2\\&+2n_{\mathrm{agg\_su}}\beta_{\mathrm{su}}\beta_{\mathrm{br}}A_{\mathrm{su\_s}}(q,r_{\mathrm{mc}})A_{\mathrm{br}}(q,R_{\mathrm{g\_br}})\frac{\sin q(r_{\mathrm{su}}+R_{\mathrm{g\_br}})}{q(r_{\mathrm{su}}+R_{\mathrm{g\_br}})}\\&+2n_{\mathrm{agg\_su}}\beta_{\mathrm{su}}\beta_{\mathrm{co}}A_{\mathrm{su\_s}}(q,r_{\mathrm{su}})A_{\mathrm{co}}(q,R_{\mathrm{g\_co}})\frac{\sin q(r_{\mathrm{su}}-R_{\mathrm{g\_co}})}{q(r_{\mathrm{su}}-R_{\mathrm{g\_co}})}\\&+2n_{\mathrm{agg\_su}}^2\beta_{\mathrm{br}}\beta_{\mathrm{co}}A_{\mathrm{br}}(q,R_{\mathrm{g\_br}})A_{\mathrm{co}}(q,R_{\mathrm{g\_co}})\frac{\sin q(r_{\mathrm{su}}+R_{\mathrm{g\_br}})}{q(r_{\mathrm{su}}+R_{\mathrm{g\_br}})}\frac{\sin q(r_{\mathrm{su}}-R_{\mathrm{g\_co}})}{q(r_{\mathrm{su}}-R_{\mathrm{g\_co}})}\end{aligned},\quad \text{(S35)}$$

where the form factor amplitude of the core block is defined as $A_{\mathrm{co}}(q,R_{\mathrm{g\_co}})=\frac{1-\exp(-q^2R_{\mathrm{g\_co}}^2)}{q^2R_{\mathrm{g\_co}}^2}$, and $R_{\mathrm{g\_co}}$ is the radius of gyration of the core block. The self-correlation term of the core block in eq S35 is described by the Debye function $F_{\mathrm{co}}(q,R_{\mathrm{g\_co}})=\frac{2\left[\exp(-q^2R_{\mathrm{g\_co}}^2)-1+q^2R_{\mathrm{g\_co}}^2\right]}{q^4R_{\mathrm{g\_co}}^4}$. The excess scattering length density of the liquid core is $\beta_{\mathrm{su}}=(\xi_{\mathrm{su}}-\xi_{\mathrm{bg}})$, where the scattering length density of the liquid core is calculated as the volume-average of the composing components as

$$\xi_{su} = \frac{\xi_{mon} f_{mon} V_{mon} + \sum_{j=1}^{n_{sol}} \xi_{solj} f_{solj} V_{solj}}{f_{mon} V_{mon} + \sum_{j=1}^{n_{sol}} f_{solj} V_{solj}}$$

. The excess scattering length density of the solvophobic block (e.g. PBzMA) of the copolymer is $\beta_{co} = (\xi_{co} - \xi_{su})$. The form factor amplitude of the BzMA-rich droplet in eq S34 is taken as

$$A_{su}(q, r_{su}) = \beta_{su} A_{su\_s}(q, r_{su}) + n_{agg\_su} \cdot \left[\beta_{br} A_{br}(q, R_{g\_br}) \frac{\sin q(r_{su} + R_{g\_br})}{q(r_{su} + R_{g\_br})} + \beta_{co} A_{co}(q, R_{g\_co}) \frac{\sin q(r_{su} - R_{g\_co})}{q(r_{su} - R_{g\_co})} \right],$$

where A_{su_s} is the BzMA-rich droplet core scattering amplitude, which is defined as

$$A_{su\_s}(q, r_{su}) = \frac{3(\sin qr_{su} - qr_{su} \cos qr_{su})}{q^3 r_{su}^3} e^{-\frac{1}{2} q^2 \sigma_t^2}$$

and the aggregation number is expressed as

$$n_{agg\_su} = x_{pol} \cdot \frac{\frac{4}{3} \pi r_{su}^3}{v_{co}}$$

. Hence the mean aggregation number is defined as $N_{agg\_su} = x_{pol} \cdot \frac{\frac{4}{3} \pi R_{su}^3}{v_{co}}$.

SAXS analysis of the initial stages of the PISA synthesis

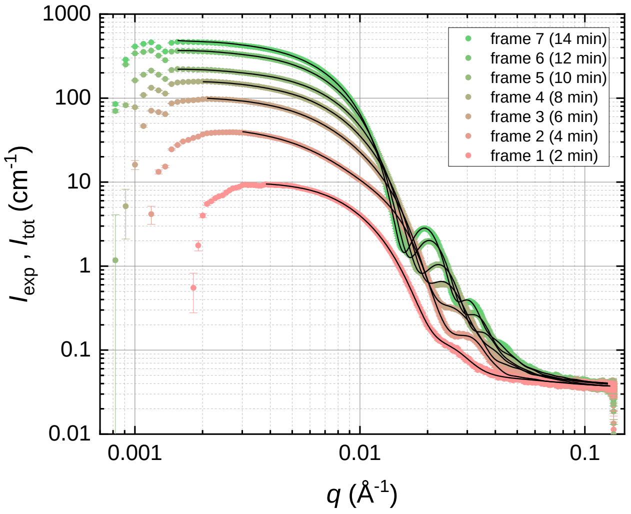

Figure S3. Experimental SAXS patterns (circles) recorded for 10 seconds every 2 min from 2 min (frame 1) to 14 min (frame 7) during the PISA synthesis of $PSMA_{31}$–$PBzMA_{2000}$ spherical nanoparticles at 90 °C in mineral oil at 10% w/w solids, with corresponding fitting curves (solid lines) produced when using the two-population SAXS model (equations S34 and S35).

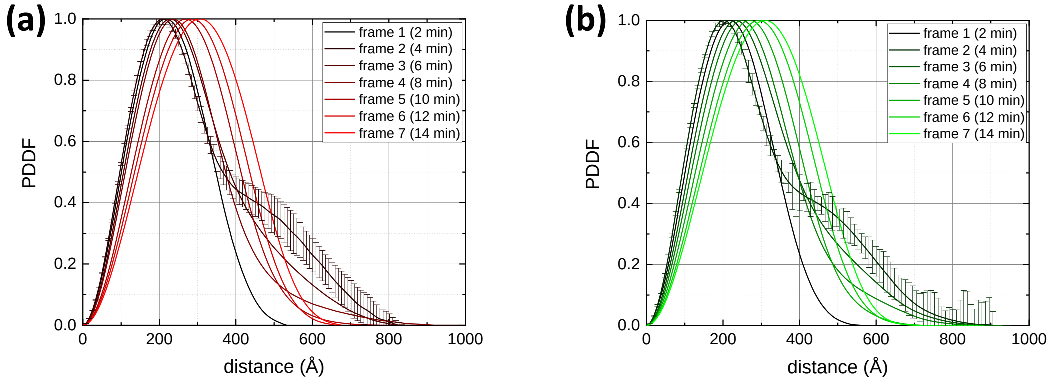

Figure S4. Normalized pair-distance distribution functions (PDDFs) calculated from (a) experimental SAXS patterns and (b) model fitting curves obtained for frames 1 to 7 (as plotted in **Figure S3**). Representative error bars are shown for the PDDF associated with frame 2.

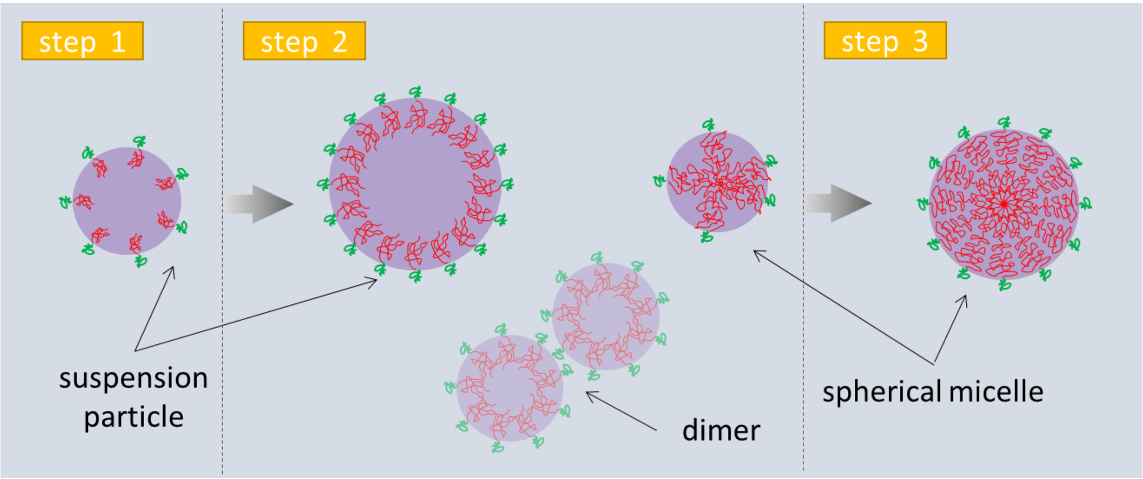

Figure S5. Schematic representation of the evolution of structure within the reaction mixture during the early stages of the PISA synthesis of $PSMA_{31}$-$PBzMA_{2000}$ spherical nanoparticles in mineral oil. Initially, (SAXS frame 1 recorded after 2 min) anomalously large spherical BzMA monomer-rich droplets stabilized by short $PSMA_{31}$-PBzMA chains are formed (see step 1). Next, (SAXS frames 2-6, recorded after 4-12 min) a binary mixture is obtained that comprises these monomer-rich droplets and nascent spherical micelles (see step 2). Potential transient species (for example, formed by fission of the large BzMA-rich droplets) are also shown. Finally, (i.e. from frame 7 recorded after 14 min to the final SAXS frame recorded after 194 min) only growing $PSMA_{31}$-PBzMA spherical micelles are present within the reaction mixture (see step 3).

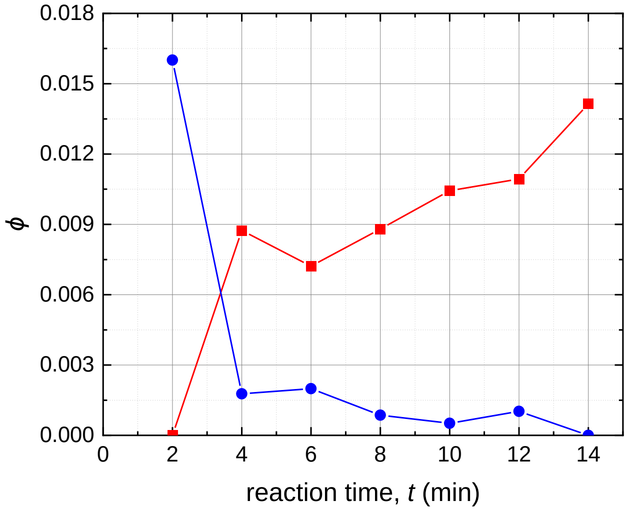

Figure S6. Variation in volume fraction, ϕ_{su}, of the anomalously large BzMA monomer-rich droplets (blue circles) and $PSMA_{31}$-PBzMA spherical micelles, ϕ_{sm}, (red squares) that are present during the early stages of PISA, as indicated by fitting the two-population micelle model to SAXS frames 1 to 7 (**Figure S3**). The lines between the data points are for guidance only.

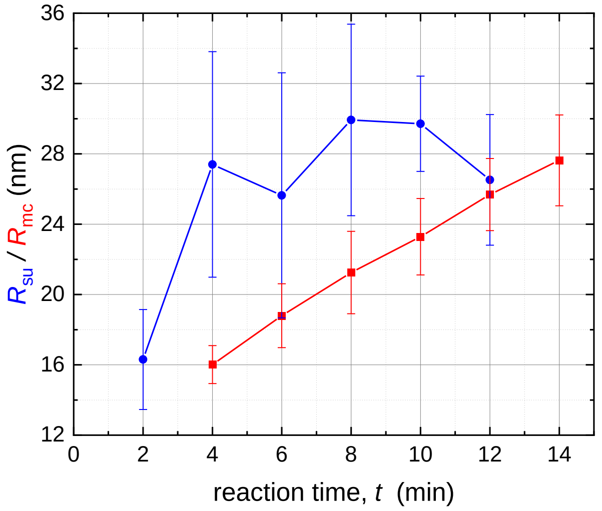

Figure S7. Variation in the mean radius of the anomalously large BzMA monomer-rich droplets, R_{su}, (blue circles) and the $PSMA_{31}$-PBzMA spherical micelles, R_{mc}, (red squares) determined during the early stages of PISA by using the two-population scattering model to fit SAXS frames 1-7 (**Figure S3**). The lines shown are merely guidance for the eye. Error bars indicate standard deviations for such radii. Notably, the mean radius of the BzMA-rich droplets always exceeds that of the spherical micelles. After 14 min, the former species disappears and only the latter species remains (see **Figure S5**, step 2).

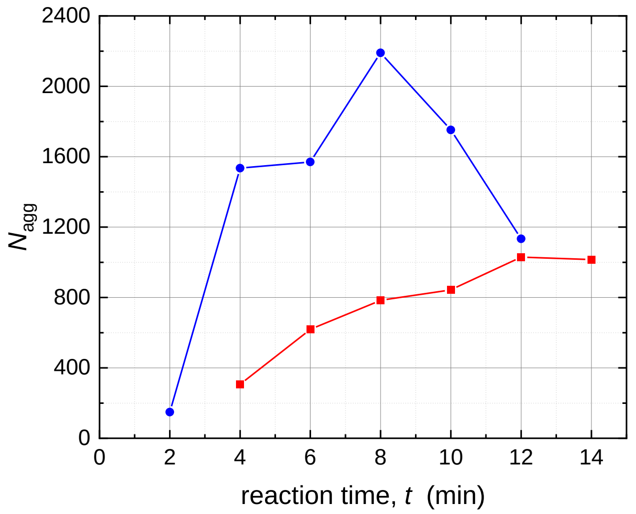

Figure S8. The mean aggregation number (N_{agg}) of $PSMA_{31}$-PBzMA copolymers stabilizing the BzMA monomer-rich droplets (circles) and forming the spherical micelles (squares) calculated by fitting the two-population model to SAXS frames recorded between 2 min (frame 1) and 14 min (frame 7) (**Figure S3**).

Calculation of the rate of BzMA polymerization after micellar nucleation during the PISA synthesis of $PSMA_{31}$-$PBzMA_{2000}$ spherical nanoparticles at 10% w/w in mineral oil

Following micellar nucleation, chain extension of the solvophobic PBzMA block involves BzMA polymerization within monomer-swollen cores (eq 2). In this case, c_{mon} is defined as the BzMA monomer molar concentrations within the spherical micelle core:[12]

$$c_{mon} = \frac{1}{N_A}\frac{1}{V_{mc\text{-}co}}\frac{n_{in\_mon}}{N_p}, \tag{S36}$$

where N_P is the total number of spherical micelles defined as $N_p = \frac{n_{br}}{N_{agg}}$, where n_{br} is the number of moles of the $PSMA_{31}$ steric stabilizer block. When combined with the expression for the mean aggregation number (eq S30), eq S36 can be rewritten as:

$$c_{mon} = \frac{1}{N_A}\frac{1}{V_{mc\text{-}co}}\frac{n_{in\_mon}}{\dfrac{n_{br}}{x_{pol}\cdot\dfrac{V_{mc\text{-}co}}{v_{co}}}} = \frac{1}{N_A}\frac{x_{pol}}{v_{co}}\cdot\frac{n_{in\_mon}}{n_{br}}. \tag{S37}$$

By defining $v_{co} = \frac{M_{co}}{\rho_{co}\cdot N_A}\cdot DP$ and $DP = \frac{m_{ini\_mon}}{M_{co}}\frac{M_{br}}{m_{br}}\cdot conv$, eq S37 can be rewritten as:

$$c_{mon} = \frac{1}{N_A}\frac{x_{pol}}{\dfrac{M_{co}}{\rho_{co}\cdot N_A}\cdot\dfrac{m_{ini\_mon}}{M_{co}}\dfrac{M_{br}}{m_{br}}\cdot conv}\cdot\frac{n_{in\_mon}}{n_{br}} = \frac{\rho_{co}}{m_{ini\_mon}}\frac{x_{pol}}{conv}\cdot n_{in\_mon}. \tag{S38}$$

Using the definitions of x_{mon} and x_{pol} (eq S18), $n_{\text{in\_mon}}$ can be expressed as $n_{\text{in\_mon}} = \frac{\rho_{\text{mon}}}{M_{\text{co}}} \cdot V_{\text{in\_mon}} = \frac{\rho_{\text{mon}}}{M_{\text{co}}} \cdot \left(f_{\text{sm}} \cdot V_{\text{co}} \cdot \frac{x_{\text{mon}}}{x_{\text{pol}}} \right)$. Thus, eq S38 could be rewritten as:

$$c_{\text{mon}} = \frac{\rho_{\text{co}}}{m_{\text{ini\_mon}}} \frac{xpol}{conv} \cdot \frac{\rho_{\text{mon}}}{M_{\text{co}}} \cdot \left(f_{\text{sm}} \cdot V_{\text{co}} \cdot \frac{x_{\text{mon}}}{x_{\text{pol}}} \right) = \frac{\rho_{\text{co}} \cdot \rho_{\text{mon}}}{m_{\text{ini\_mon}}} \frac{f_{\text{sm}} \cdot V_{\text{co}}}{M_{\text{co}} \cdot conv} \cdot x_{\text{mon}} . \quad \text{(S39)}$$

Following the definition of V_{co} (eq S7), eq S39 can be rewritten as:

$$c_{\text{mon}} = \frac{\rho_{\text{co}} \cdot \rho_{\text{mon}}}{m_{\text{ini\_mon}}} \frac{f_{\text{sm}} \cdot conv \cdot \frac{m_{\text{ini\_mon}}}{\rho_{\text{co}}}}{M_{\text{co}} \cdot conv} \cdot x_{\text{mon}} = f_{\text{sm}} \cdot \frac{\rho_{\text{mon}}}{M_{\text{co}}} \cdot x_{\text{mon}} . \quad \text{(S40)}$$

Since x_{mon} is the only time-dependent variable in eq S7, the time-dependent c_{mon} can be expressed as:

$$c_{\text{mon}}(t) = f_{\text{sm}} \cdot \frac{\rho_{\text{mon}}}{M_{\text{co}}} \cdot x_{\text{mon}}(t) . \quad \text{(S41)}$$

The radical molar concentration within the spherical micelle core, $c_{\text{P}\cdot}(t)$, is defined as the spherical micelle number density multiplied by the number of radicals within the spherical micelle core:

$$c_{\text{P}\cdot}(t) = f_{\text{sm}} \cdot [I\cdot](t) . \quad \text{(S42)}$$

Here $[I\cdot](t)$ is the time-dependent total radical concentration in the system defined as $\sqrt{\frac{k_{\text{d}}}{k_{\text{t}}} \frac{f_{\text{in}} n_{\text{T21s}}}{V_{\text{tot}}} e^{-k_{\text{d}} \cdot t}}$, where k_{d} is the decomposition rate coefficient of the initiator, k_{t} is the termination rate coefficient, n_{T21s} is the initial number of moles of T21s initiator, and f_{in} is the initiator efficiency[1a]. It has to be noted that after the micelle formation when all copolymer molecules localised in the particles $f_{\text{sm}} = 1$ and, therefore, at this stage of the synthesis (**Stage 4** onwards, **Figure 1**) $c_{\text{P}\cdot}(t) = [I\cdot](t)$.

It should be noted that the high-brilliance synchrotron X-ray beam leads to a significantly faster rate of polymerization (**Figure 4**), which is attributed to the generation of a further radical flux in addition to that provided by the initiator. Under such conditions, the reduction in the initiator concentration can be neglected and k_{d} can be assumed to be constant because continuous X-ray irradiation ensures a constant supply of radicals. Accordingly, eq. 3 (see main manuscript) can be rewritten as

$$R_{\text{p}}(t) = k_p \cdot \frac{\rho_{\text{mon}}}{M_{\text{co}}} \cdot f_{\text{sm}}^{\ 2} \cdot \sqrt{\frac{k_{\text{d}}}{k_{\text{t}}} \frac{n_{\text{T21s}}}{V_{\text{tot}}}} \cdot x_{\text{mon}}(t) = k_p^{'} \cdot x_{\text{mon}}(t), \quad \text{(S43)}$$

This modified equation produces an even better linear correlation between x_{mon} and $R_{\text{p\_exp}}$ (compare **Figure S9** with **Figure 7**) and only a slightly different k_{p}' of 4.46×10^{-2} min^{-1} (**Figure S9**) compared to that associated with **Figure 7** ($k_{\text{p}}' = 4.89 \times 10^{-2}$ min^{-1}). By analogy to the initial analysis using eq. 3, the initiator decomposition rate constant k_{d} was taken to be equal to that known for T21s initiator at 90 °C (1.92×10^{-4} s^{-1}). However, the true value is likely to be higher owing to additional radicals generated by the X-ray beam. Thus, the calculated new k_{p}' is best considered as a relative value that

requires correction for the additional radical flux generated by X-ray radiation of the sample, which is unknown.

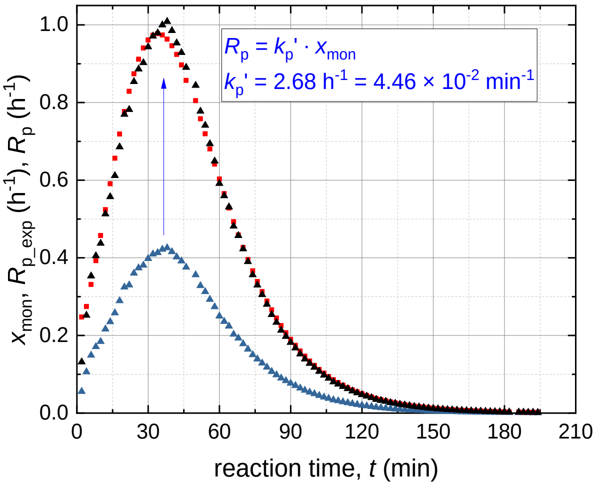

Figure S9. Time-dependence of x_{mon} and R_{p_exp} obtained from SAXS results (blue triangles and red squares, respectively). The x_{mon} curve matches the R_{p_exp} curve if multiplied by k_p' (see black triangles representing $R_p = k_p' \cdot x_{mon}$). For the sake of clarity, h^{-1} unit was used for R_{p_exp} and R_p.

The calculated k_p' values can be used to estimate the propagation rate coefficient k_p. According to eq 3 (see main manuscript) $k_p = k_p' \cdot c_{ini\_mon} \cdot \frac{M_{co}}{\rho_{mon}} \cdot \frac{1}{f_{sm}^{\ 2}} \cdot \sqrt{\frac{k_t}{k_d} \frac{V_{tot}}{f_{in} n_{T21s}}}$. For the case of exponential decay of the initiator concentration it was found that $k_p' = 4.89 \times 10^{-2}$ min^{-1} (**Figure 7**). Assuming f_{in} = 1 and the typical range of k_t as $10^6 - 10^8$ L· mol^{-1}· s^{-1} [13], the minimum and maximum values of k_p are calculated to be:

$$k_{p\ max} = \frac{0.00489\ s^{-1}}{60} \cdot \frac{0.217\ g}{176\ g/mol \times 0.00277\ L} \cdot \frac{176\ g/mol}{960\ g/L} \cdot \frac{1}{1} \cdot \sqrt{\frac{1\times10^{8}\ L\cdot mol\cdot s^{-1}}{1.92\times10^{-4}\ s^{-1}} \frac{0.00277\ L}{1\times\left(\frac{1}{5}\times\frac{0.00663\ g}{10478\ g/mol}\right)}} = 7.09\times10^{3}\ L\cdot mol^{-1}\cdot s^{-1}$$

$$k_{p\ min} = \frac{0.00489\ s^{-1}}{60} \cdot \frac{0.217\ g}{176\ g/mol \times 0.00277\ L} \cdot \frac{176\ g/mol}{960\ g/L} \cdot \frac{1}{1} \cdot \sqrt{\frac{1\times10^{6}\ L\cdot mol\cdot s^{-1}}{1.92\times10^{-4}\ s^{-1}} \frac{0.00277\ L}{1\times\left(\frac{1}{5}\times\frac{0.00663\ g}{10478\ g/mol}\right)}} = 7.09\times10^{2}\ L\cdot mol^{-1}\cdot s^{-1}$$

For the case of continuous flux of radicals generated by X-ray radiation it was found that $k_p' = 4.46 \times 10^{-2}$ min^{-1} (**Figure S9**). Thus, considering this condition of the reaction $k_{p\ min} = 6.47 \times 10^{2}$ L· mol^{-1}· s^{-1} and $k_{p\ max} = 6.47 \times 10^{3}$ L· mol^{-1}· s^{-1}. The range of k_p values estimated for both cases is consistent with a k_p value of 3.0×10^{3} L· mol^{-1}· s^{-1} determined for the bulk polymerization of BzMA at 90 °C.[14]

Experimental Methods

The PISA synthesis of poly(stearyl methacrylate)-poly(benzyl methacrylate) block copolymer ($PSMA_{31}$-$PBzMA_{2000}$) diblock copolymer spheres via RAFT dispersion polymerization of BzMA using a $PSMA_{31}$ precursor in mineral oil at 90 °C (see **Scheme 1**) has been described elsewhere, along with the experimental setup for simultaneous TR-SAXS data collection.[15] Briefly, BzMA monomer (0.217 g; 1.23 mmol), tert-butyl peroxy-2-ethylhexanoate (T21s) initiator (0.274 mg; 0.127 µmol; dissolved at 10.0 % v/v in mineral oil) and the $PSMA_{31}$ precursor (6.63 mg; 0.633 µmol; $PSMA_{31}$/T21s molar

ratio = 5.0; target PBzMA DP = 2000) were dissolved in mineral oil (2.02 g). The reaction mixture was sealed in a 10 mL round-bottomed flask and purged with nitrogen gas for 30 min before an aliquot of the deoxygenated solution was transferred into a 2.0 mm diameter glass capillary for the TR-SAXS study (WJM-Glas, Berlin, Germany). The capillary was then sealed in order to prevent oxygen ingress before placing in a brass holding stage mounted on a synchrotron X-ray beamline (station I22, Diamond Light Source, Didcot, UK).[16] At the beginning of the SAXS data collection the brass stage was connected, via a pneumatic switch, to a water circulator pre-heated to 90 °C to initiate the polymerization. After completing the SAXS measurements, *postmortem* analysis of the reaction mixture was undertaken. The final BzMA conversion was determined to be 98% by ^{1}H NMR spectroscopy. Unfortunately, the dispersity (M_w/M_n) value for the diblock copolymer chains synthesised during the SAXS experiment cannot be measured because the glass capillary cell does not hold a sufficiently large sample volume. However, gel permeation chromatography (GPC) analysis of the same diblock copolymer using an identical PISA formulation prepared on a laboratory scale in the absence of X-rays indicated a number-average molecular weight (M_n) of 140 200 g·mol^{-1} and an M_w/M_n of 1.91.[15]

SAXS patterns were recorded for 10 seconds duration every 2 min over the reaction time of 194 min (97 frames in total) using monochromatic X-ray radiation (λ = 1.24 Å, q ranges from 0.0015 to 0.13 Å^{-1}) and a 2D Pilatus 2M pixel detector (Dectris, Switzerland). Thus, the sample was exposed to X-ray radiation for 10 seconds every 2 minutes. 2D SAXS patterns were reduced to 1D SAXS profiles (I_{exp}, see **Figure 3**) by integration, with calibration to absolute intensity achieved using the SAXS pattern recorded for deionized water (assuming that the differential scattering cross-section of water at 21 °C is 0.0162 cm^{-1}) using Dawn software supplied by Diamond Light Source.[17]

Prior to fitting the SAXS model described by eq S1, the experimental 1D SAXS profiles were further corrected by subtracting the background scattering recorded for an empty glass capillary heated to 90 °C. Clearly, the solvent composition changes during PISA as the BzMA monomer is converted into the solvophobic PBzMA chains. Moreover, some fraction of the unreacted BzMA monomer (and possibly also some solvent molecules) becomes localized within the growing micelles (**Figure 1**). Thus, the solvent background scattering term, I_{bg}, is included in the SAXS model (eq S1). Empirically, the I_{bg} dependence on the BzMA monomer concentration (w_{mon}) can be interpolated by a cubic polynomial (equations S14 and S15) fitted to the X-ray scattering intensity measured for a series of binary mixtures of mineral oil and BzMA by SAXS (**Figure S2**). The resulting analytical expression for I_{bg} was incorporated into the SAXS model (eq S1). The densities of BzMA and mineral oil at 90 °C (0.975 g·cm^{-3} and 0.785 g·cm^{-3}, respectively) were determined by the oscillating U-tube method using a DMA5000 density meter (Anton Paar, Graz, Austria). Using a series of solutions of differing $PSMA_{31}$ concentration, the density of $PSMA_{31}$ in mineral oil at 90 °C was determined to be 0.890 g·cm^{-3} by the same method. The density of PBzMA at 90 °C was estimated to be 1.14 g·cm^{-3} by using a helium pycnometer to determine the density of PBzMA powder at 20 °C and assuming that this homopolymer has the same thermal expansion coefficient as poly(methyl methacrylate).[18],[19] The X-ray scattering length densities of BzMA, $PSMA_{31}$, PBzMA and mineral oil, required for SAXS analysis (equations S6 and S7), were calculated from the corresponding chemical composition and mass density of each component at 90 °C to be 8.82 × 10^{10} cm^{-2}, 9.17 × 10^{10} cm^{-2}, 1.03 × 10^{11} cm^{-2} and 7.63 × 10^{10} cm^{-2}, respectively.[15] All four components (i.e. monomer, solvent, PSMA precursor and the growing PBzMA chains) have a reasonably high scattering length density contrast with each other, which facilitates the data analysis. Relative differences between the mass densities of these four components is similarly beneficial. Clearly, if the scattering length density and/or mass density

of two or more components are too close to each other, then SAXS may not be sufficiently sensitive to discriminate between them.

Calculations and fitting of the SAXS data (**Figure 3**) were performed using *Irena,*[20] a macro package for small-angle scattering (SAS) data analysis developed for the commercial *Igor Pro* software. The SAXS model (eq S1) was programmed using a user function option in the *Irena* SAS macros. Ten independent parameters were used to fit the experimental SAXS patterns (**Table S1**). To program relationships linked to multiple populations of scattering objects and solvent background scattering, five of the SAXS model parameters (ϕ_{sm}, ϕ_{pc}, *conv*, x_{mon} and x_{sol}) were assigned as global variables in the coded *Igor Pro* macro.

References

[1] a) S. Perrier, *Macromolecules* **2017**, *50*, 7433-7447; b) M. J. Derry, L. A. Fielding, S. P. Armes, *Progress in Polymer Science* **2016**, *52*, 1-18; c) N. J. Warren, S. P. Armes, *Journal of the American Chemical Society* **2014**, *136*, 10174-10185.

[2] E. E. Brotherton, F. L. Hatton, A. A. Cockram, M. J. Derry, A. Czajka, E. J. Cornel, P. D. Topham, O. O. Mykhaylyk, S. P. Armes, *Journal of the American Chemical Society* **2019**, *141*, 13664-13675.

[3] B. Hammouda, in *Polymer Characteristics*, Springer, **1993**, pp. 87-133.

[4] J. S. Pedersen, M. C. Gerstenberg, *Macromolecules* **1996**, *29*, 1363-1365.

[5] a) R. Lund, L. Willner, D. Richter, E. E. Dormidontova, *Macromolecules* **2006**, *39*, 4566-4575; b) R. Lund, L. Willner, J. Stellbrink, P. Lindner, D. Richter, *Physical review letters* **2006**, *96*, 068302; c) R. Lund, L. Willner, M. Monkenbusch, P. Panine, T. Narayanan, J. Colmenero, D. Richter, *Physical Review Letters* **2009**, *102*, 188301; d) R. Lund, L. Willner, P. Lindner, D. Richter, *Macromolecules* **2009**, *42*, 2686-2695.

[6] J. S. Pedersen, *The Journal of Chemical Physics* **2001**, *114*, 2839-2846.

[7] A. Blanazs, J. Madsen, G. Battaglia, A. J. Ryan, S. P. Armes, *Journal of the American Chemical Society* **2011**, *133*, 16581-16587.

[8] E. J. Cornel, S. van Meurs, T. Smith, P. S. O'Hora, S. P. Armes, *Journal of the American Chemical Society* **2018**, *140*, 12980-12988.

[9] D. J. Kinning, E. L. Thomas, *Macromolecules* **1984**, *17*, 1712-1718.

[10] J. S. Pedersen, C. Svaneborg, K. Almdal, I. W. Hamley, R. N. Young, *Macromolecules* **2003**, *36*, 416-433.

[11] J. S. Pedersen, *Advances in Colloid and Interface Science* **1997**, *70*, 171-210.

[12] P. A. Lovell, M. S. El-Aasser, *Emulsion Polymerization and Emulsion Polymers*, Wiley, **1997**.

[13] G. Odian, in *Principles of Polymerization*, John Wiley & Sons, Inc., Hoboken, New Jersey, **2004**, pp. 198-349.

[14] S. Beuermann, M. Buback, T. P. Davis, N. García, R. G. Gilbert, R. A. Hutchinson, A. Kajiwara, M. Kamachi, I. Lacík, G. T. Russell, *Macromolecular Chemistry and Physics* **2003**, *204*, 1338-1350.

[15] M. J. Derry, L. A. Fielding, N. J. Warren, C. J. Mable, A. J. Smith, O. O. Mykhaylyk, S. P. Armes, *Chemical Science* **2016**, *7*, 5078-5090.

[16] A. Smith, S. Alcock, L. Davidson, J. Emmins, J. Hiller Bardsley, P. Holloway, M. Malfois, A. Marshall, C. Pizzey, S. Rogers, *Journal of synchrotron radiation* **2021**, *28*, 939-947.

[17] M. Basham, J. Filik, M. T. Wharmby, P. C. Chang, B. El Kassaby, M. Gerring, J. Aishima, K. Levik, B. C. Pulford, I. Sikharulidze, *Journal of synchrotron radiation* **2015**, *22*, 853-858.

[18] J. E. Mark, *Physical Properties of Polymers Handbook*, Springer New York, **2007**.

[19] L. A. Fielding, J. A. Lane, M. J. Derry, O. O. Mykhaylyk, S. P. Armes, *Journal of the American Chemical Society* **2014**, *136*, 5790-5798.

[20] J. Ilavsky, P. R. Jemian, *Journal of Applied Crystallography* **2009**, *42*, 347-353.
